# The quality of SIV-specific fCD8 T cells limits SIV RNA production in Tfh cells during antiretroviral therapy

**DOI:** 10.1128/jvi.00812-24

**Published:** 2024-12-06

**Authors:** Shokichi Takahama, Ayaka Washizaki, Tomotaka Okamura, Shingo Kitamura, Takuto Nogimori, Yorifumi Satou, Yasuhiro Yasutomi, Tomokazu Yoshinaga, Takuya Yamamoto

**Affiliations:** 1 Laboratory of Precision Immunology, Center for Intractable Diseases and ImmunoGenomics, National Institutes of Biomedical Innovation, Health and Nutrition, Osaka, Japan; 2 Laboratory of Immunoregulation and Vaccine Research, Tsukuba Primate Research Center, National Institutes of Biomedical Innovation, Health and Nutrition, Tsukuba, Ibaraki, Japan; 3 Laboratory for Drug Discovery and Disease Research, Shionogi & Co., Ltd 73768 , Toyonaka, Osaka, Japan; 4 Division of Genomics and Transcriptomics, Joint Research Center for Human Retrovirus Infection, Kumamoto University 624497 , Kumamoto, Japan; 5 Department of Virology and Immunology, Graduate School of Medicine, Osaka University 595206 , Osaka, Japan; 6 Laboratory of Aging and Immune Regulation, Graduate School of Pharmaceutical Sciences, Osaka University 595206 , Osaka, Japan; 7 The Research Institute for Microbial Diseases, Osaka University, Osaka, Japan; Icahn School of Medicine at Mount Sinai, New York, New York, USA

**Keywords:** SIV, SIV-specific fCD8 T cells, cART, latently-SIV-infected reservoirs, non-human primates

## Abstract

The attack and defense of infected cells and cytotoxic CD8 T cells occur in germinal centers in lymphoid tissue in chronic persistent HIV/SIV infection. Latently infected cells, the therapeutic target of HIV infection, accumulate in follicular helper T (Tfh) cells in lymphoid tissue; the impact of HIV-specific follicular CD8 (fCD8) T cells in lymphoid tissue on the latently infected cells remains unknown. We infected 15 cynomolgus macaques with SIVmac239 and examined the contribution of SIV-Gag-specific fCD8 T cells, defined by activation-induced markers (AIMs), to SIV-infected cells. Eight out of the 15 infected macaques served as progressors; a chronic phase combination antiretroviral therapy (cART) model was established for the eight macaques (progressors) with chronic persistent infection status, wherein cART was started in the chronic phase and discontinued after 27 weeks. Seven macaques that naturally controlled the viremia served as natural controllers. The frequency of SIV-Gag-specific fCD8 T cells was inversely correlated with the amount of cell-associated SIV-*gag* RNA in the Tfh only under cART or in the controllers but not in untreated progressors. scRNA-seq of SIV-Gag-specific fCD8 T cells in various conditions revealed that the gene expression pattern of SIV-Gag-specific fCD8 T cells in the controllers was closer to that of those under cART than the untreated progressors. Comparing the SIV-Gag-specific fCD8 T cells of those under cART to the controllers revealed their more exhausted and immunosenescent nature under cART. Improving the HIV/SIV-specific fCD8 T cells under cART by targeting those pathways might contribute to the development of potential curative strategies.

**IMPORTANCE:**

We infected cynomolgus macaques with SIVmac239 to establish an SIV-chronically infected cART model. We performed an in-depth characterization of Tfh and fCD8 T cells in three conditions—chronic stage of untreated, cART-treated, and natural controller cynomolgus macaques—by combining tissue section analysis and single-cell analyses of sorted cells. We revealed the inverse relationship between Tfh infection and SIV-Gag-specific fCD8 T cell frequencies as observed in HIV-infected individuals, thereby establishing the cynomolgus macaque as a relevant animal model to study the determinants of HIV/SIV persistence in lymphoid tissue. Additionally, scRNA-seq analysis of SIV-Gag-specific fCD8 T cells revealed an enrichment of exhausted or senescent transcriptomic signatures under cART. These data will provide the basic insights into virus-host CD8 T cell interactions, particularly within the follicular region, during latent HIV infection under ART.

## INTRODUCTION

With the widespread acceptance of combined antiretroviral therapy (cART), it has become more common to control the onset of acquired immunodeficiency syndrome (AIDS), even if a person is infected with the human immunodeficiency virus (HIV). However, cART does not fully restore the immune system, and HIV-infected individuals receiving cART are considered people living with HIV (PLWH). PLWH have a higher risk of AIDS-related illness and death than people of the same age without HIV (1–6). In PLWH undergoing cART, the number of latent HIV-infected cells tends to decrease gradually over time as viral replication is suppressed. However, its half-life is extremely long (7, 8), and a certain number of latently infected cells in the body always exist. Thus, by interrupting cART, viral replication is activated. Therefore, to create a cART-free state, that is, to cure HIV infection, it is essential to eliminate these latently infected cells. It has been suggested that the reactivation of latently infected cells, induction of cell death by the cytopathic effect (CPE), and potent activation of the anti-HIV-1 immune response are important for the realization of an HIV-1 cure (9). However, current treatments alone have not been able to induce them simultaneously.

During HIV infection, high levels of viral DNA have been detected in lymphoid tissues under various conditions, including cART (10, 11). Latently infected cells are thought to be localized in the lymphoid tissue, particularly in follicular helper CD4 T cells (Tfh cells) (3, 12). Gene expression analysis has revealed important properties of Tfh cells in simian immunodeficiency virus (SIV)-infected and uninfected macaques, such as rhesus macaques, which exhibit a gene profile completely different from non-Tfh cells. Tfh is a target cell for SIV infection, and considerable changes in Tfh gene expression due to SIV infection and accumulation of Tfh in the chronic phase are observed in some macaques (12). Furthermore, analysis of the SIV-infected elite controllers (EC) showed that SIV replication is concentrated in Tfh cells in the follicles of lymph nodes (LNs) and that there are few CD8 T cells in the germinal center (GC) (13). In addition, CD8 T cells that recognize the highly immunogenic Gag CM9 epitope are disproportionately located outside the follicular LNs (14). This has also been reported in patients receiving cART (15).

CD8 T-cell depletion experiments using MT807R1 (CD8-depletion antibody) in rhesus macaques have shown that CD8 T cells contribute to viral suppression under cART (16). Recent analyses of human and monkey-derived lymphoid tissue have shown that the accumulation of follicular CD8 T cells (fCD8 T cells) in the chronic phase in the GC, especially those defined by CXCR5-positive CCR7 negative, may be important for viral control (17–20). In fCD8 T cells, granzyme and perforin levels, which are involved in cytotoxicity, remain high (18). Moreover, CXCR5-positive multifunctional Gag-specific fCD8 T cells were frequently observed in low viral groups in various organs, including the secondary lymphoid tissue of chronically and persistently infected rhesus macaques, suggesting that tissue localization and multifunctionality are important factors in determining the properties of effective CD8 T cells (21). However, whether quantitative and qualitative differences in fCD8 T cells affect latent cell regulation in the chronic persistent infection phase or changes in the plasma viral load after cART interruption have not yet been directly demonstrated.

Although rhesus macaques are the most widely used animal models essential for tissue analysis, cynomolgus macaques are also recognized as HIV animal models (22–24). In addition, the limited MHC haplotype of the cynomolgus macaques from Mauritius has been reported to be advantageous over the rhesus for T-cell analyses (25). However, to date, no SIV chronic persistent infection cART model using cynomolgus macaques has been reported.

In this study, we developed a cART protocol initiated during the chronic phase of infection to control the plasma viral load of cynomolgus macaques infected with SIV for a long time below the detection limit. To clarify the contribution of the SIV-specific fCD8 T-cell response in lymphoid tissues that potentially eliminates latently infected cells, we performed a mutual analysis between virus-infected cells and fCD8 T cells at the tissue level in an SIV-chronically infected cART model.

## RESULTS

### Accumulation of latently infected cells in Tfh in an SIV-chronically infected cynomolgus macaque model

Fifteen cynomolgus macaques were transrectally infected with SIVmac239 up to three times at 5 × 10^4^ TCID50. The week before the plasma viral load (PVL) was detected was defined as 0 week of infection. Peak PVL was observed from week 1 to week 6 [median, 1.5 weeks post-infection (wpi)] after SIV infection in all macaques. (Fig. S1A).

**Fig 1:**
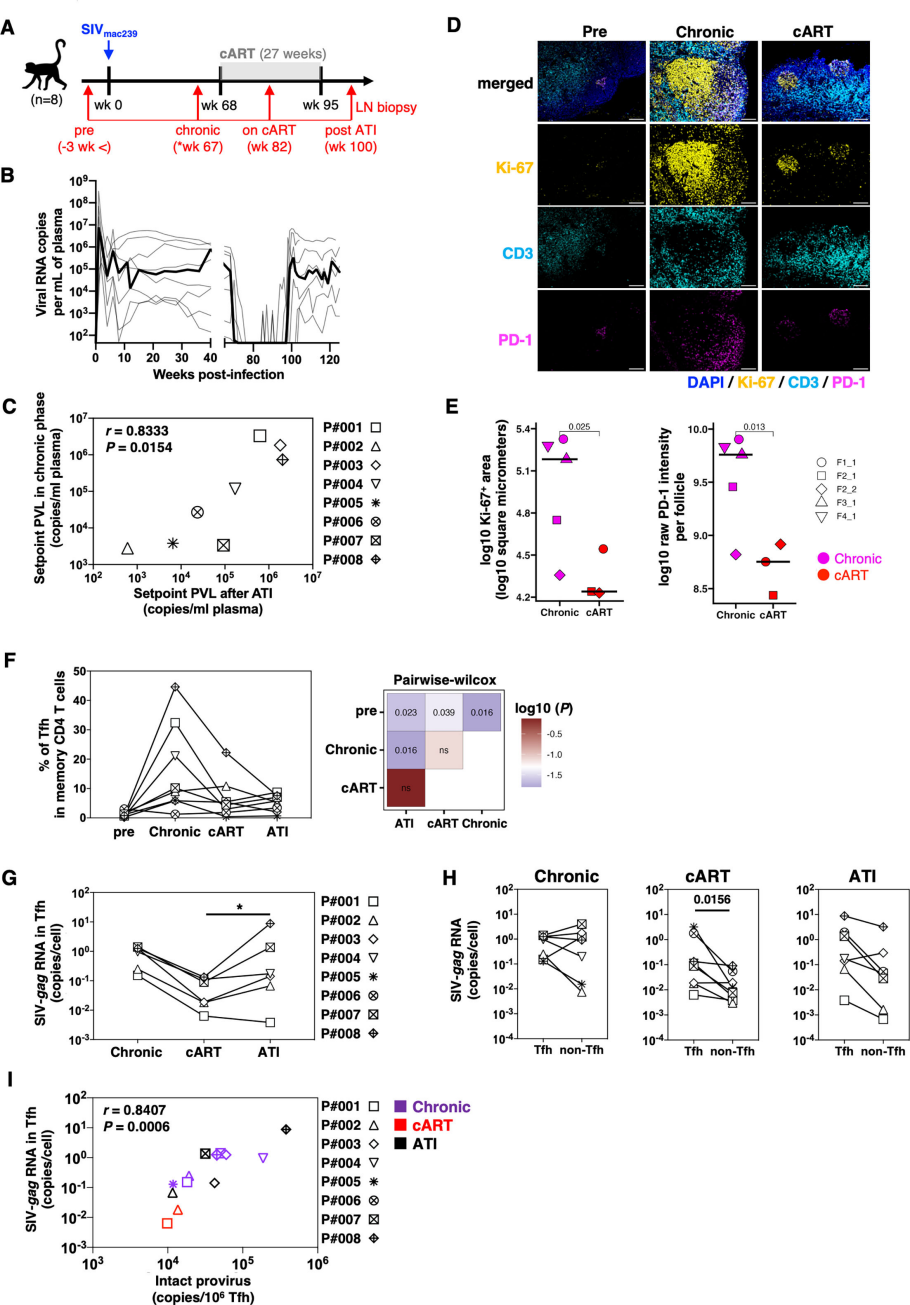
Change in Tfh frequency during the course of SIV infection. (**A**) Schematic depiction of the course of SIV infection, treatment, and its interruption. The gray box shows the cART period. Red arrows indicate the timing of LN biopsy. (**B**) Plasma viral RNA copies of progressors (*N* = 8) are plotted on the graph. Plasma viral RNA was measured by qRT-PCR. The black line shows the median. (**C**) Scatter plot depicts the correlation of setpoint plasma viral load (PVL) at the chronic phase and after ATI. Setpoint PVLs during the chronic phase were calculated as the median of PVLs measured from the point of peak VLs (around week 12 up to week 68). Setpoint PVLs after ATI were calculated as the median of VLs measured from the post-peak period up to week 125. Statistical significance was calculated using Spearman correlation analysis. (**D**) Representative immunohistochemistry data showing follicle structure in lymph node and Tfh. (**E**) Ki-67^+^ area per follicle (left) and fluorescent intensity of PD-1 signal per follicle (right) are shown. Symbols indicate the microscopic field and the follicles observed in one macaque. Crossbars indicate the median value. *P*-values calculated using the Student’s *t*-test are shown. (**F**) Time course of the frequency of Tfh in memory CD4^+^ T cells was analyzed by cytometry (left). *P*-values calculated using the pairwise Wilcoxon test are shown as a heatmap (right). (**G**) Time course of SIV-*gag* RNA in Tfh was measured by qRT-PCR. SIV-*gag* RNA in Tfh and Non-Tfh were compared. *P*-values calculated using the Wilcoxon matched-pairs signed-rank test are shown, *<0.05 (**H**) SIV-*gag* RNA copies in Tfh and Non-Tfh at the chronic phase were analyzed by qRT-PCR. *P*-values calculated using the Wilcoxon matched-pairs signed-rank test are shown. *<0.05. (**I**) SIV-*gag* RNA copies in Tfh were measured by qRT-PCR. Intact provirus was measured by IPDA. The correlation was calculated using Spearman’s rank correlation test. Tfh, follicular helper T cells; cART, combined antiretroviral therapy; IPDA, Intact proviral DNA assay; LN, lymph node.

Previous studies have shown that latently infected cells accumulate in the Tfh of SIVmac239-infected rhesus macaques and HIV-infected individuals, maintaining a chronic persistent infection state (13, 15). To clarify whether Tfh was the main latently infected cell accumulation site in the SIVmac239-infected cynomolgus macaques used in this study, LN biopsies were performed longitudinally as shown in Fig. 1A, and total lymphocytes were purified. Subsequently, the Tfh-cell fractions of PD-1^high^, CXCR5^high^, and other non-Tfh-cell fractions in memory CD4^+^ T cells were separated using a cell sorter (Fig. S2A and B). Cellular RNA was extracted from both Tfh and Non-Tfh cells; the cell-associated SIV-*gag* RNA copy numbers were measured by quantitative reverse transcriptase polymerase chain reaction (qRT-PCR), and the values were normalized to the sorted cell count. Although the cell-associated SIV-*gag* copy number in Tfh cells during the untreated chronic phase tended to be higher than that in the non-Tfh group, the difference was not statistically significant (Fig. S1B). This result, consistent with previous studies on the untreated rhesus chronic persistent infection phase (12), suggests that there is no accumulation of cell-associated SIV-*gag*-producing cells in the Tfh during the untreated chronic persistent infection phase in SIVmac239-infected cynomolgus macaques. It also suggested that the macaques could be classified into a group with a clear setpoint (*N* = 8) and a group with a viral load below the detection limit (*N* = 7). Since 8 of the 15 macaques maintained a chronic and persistent state of infection and the average PVL setpoint of 8 macaques was 2.5 × 10^5^ copies/mL (range: 10^3^–10^7^ copies/mL), we referred to these macaques as the progressor group. Then, we investigated the relationship between fCD8 T cells and viral replication control in lymphoid tissue in these progressors. In the progressor group, cART [dolutegravir (DTG)/emtricitabine (FTC)/tenofovir (TFV)] was initiated at 68 wpi, which is the stage of chronic SIV infection. The cART was administered subcutaneously once daily. cART efficiently suppressed viral growth, and the PVL was maintained below the detection limit. Then, we discontinued cART, to induce the so-called analytical treatment interruption (ATI), after 27 weeks of treatment and observed a rebound in PVL in all eight macaques (Fig. 1A and B). Post-rebound PVL setpoints were also positively correlated with the PVL setpoints during chronic infections (Fig. 1C). These results suggest that cART in our SIV-infected cynomolgus macaque model can efficiently suppress viral proliferation when initiated during the chronic SIV infection phase, as observed in HIV-infected individuals.

Next, since previous studies on SIV-infected cART models reported that Tfh accumulated during the chronic infection phase (12) and decreased with cART (13), we decided to verify whether similar infection kinetics could be observed in our chronic-phase cART model of SIV-infected cynomolgus macaques. First, the dynamics of follicular structure in LN imaging and the intrafollicular distribution of Tfh were verified by immunofluorescence staining of lymphoid tissue sections obtained from lymphoid tissue over time before and after SIV infection. Similar to previous reports on HIV-infected individuals and SIV-infected rhesus macaques, follicles detected as accumulated structures of Ki-67-positive cells enlarged during the chronic infection phase and shrank under cART (Fig. 1D; Fig. S3A and B). These longitudinal changes in follicular size were further supported by the quantification of the Ki-67^+^ follicle area observed in different fields of the histochemical slices (Fig. 1E, left). PD-1^+^/CD3^+^ cells were localized in the follicle, suggesting the accumulation of Tfh in the follicle (Fig. 1D; Fig. S3A and C). The fluorescence intensity of PD-1 signals, which were negligible in pre-infection, in follicles was upregulated in the chronic phase and reduced under cART (Fig. 1E, right), further supporting the accumulation and reduction of Tfh in the follicles in the chronic phase and under cART, respectively. Moreover, quantification by flow cytometry revealed that the proportion of Tfh in CD4^+^ memory T cells increased after SIV infection, as in previous reports, and tended to decrease after cART (Fig. 1F). In addition, the number of cell-associated SIV-*gag* copies per Tfh-cell decreased with cART compared to that in the chronic infection phase and increased significantly after cART interruption (Fig. 1G). Furthermore, the amount of cell-associated SIV-*gag* was significantly higher in Tfh than in non-Tfh only under cART but not in the chronic phase or after ATI (Fig. 1H).

The intact provirus number measured by the intact proviral DNA assay (IPDA) (26–29) significantly correlated with the cell-associated SIV-*gag* copy number measured by qRT-PCR (Fig. 1I), indicating that the cell-associated SIV-*gag* copy number correlated with the amount of intact provirus which have the potential to produce infective virus. Notably, the measurement of SIVmac239 *gag* mRNA copy number by qRT-PCR was more sensitive than that of an intact provirus measurement using the IPDA method. Thus, in subsequent analyses, the SIVmac239 cell-associated SIV-*gag* copy number was used as the probe for potential reservoir size. Together, these results demonstrate that our chronic-phase cART model of SIV-infected cynomolgus macaques also controls PVL to below the detection limit by initiating cART in the chronic phase of viral infection, as in humans and rhesus macaques and that Tfh cells are major reservoirs under cART.

### SIV-Gag-specific Activation-induced markers (AIM)^+^ fCD8 T cells contribute to the control of virus replication in Tfh under cART

**Fig 2:**
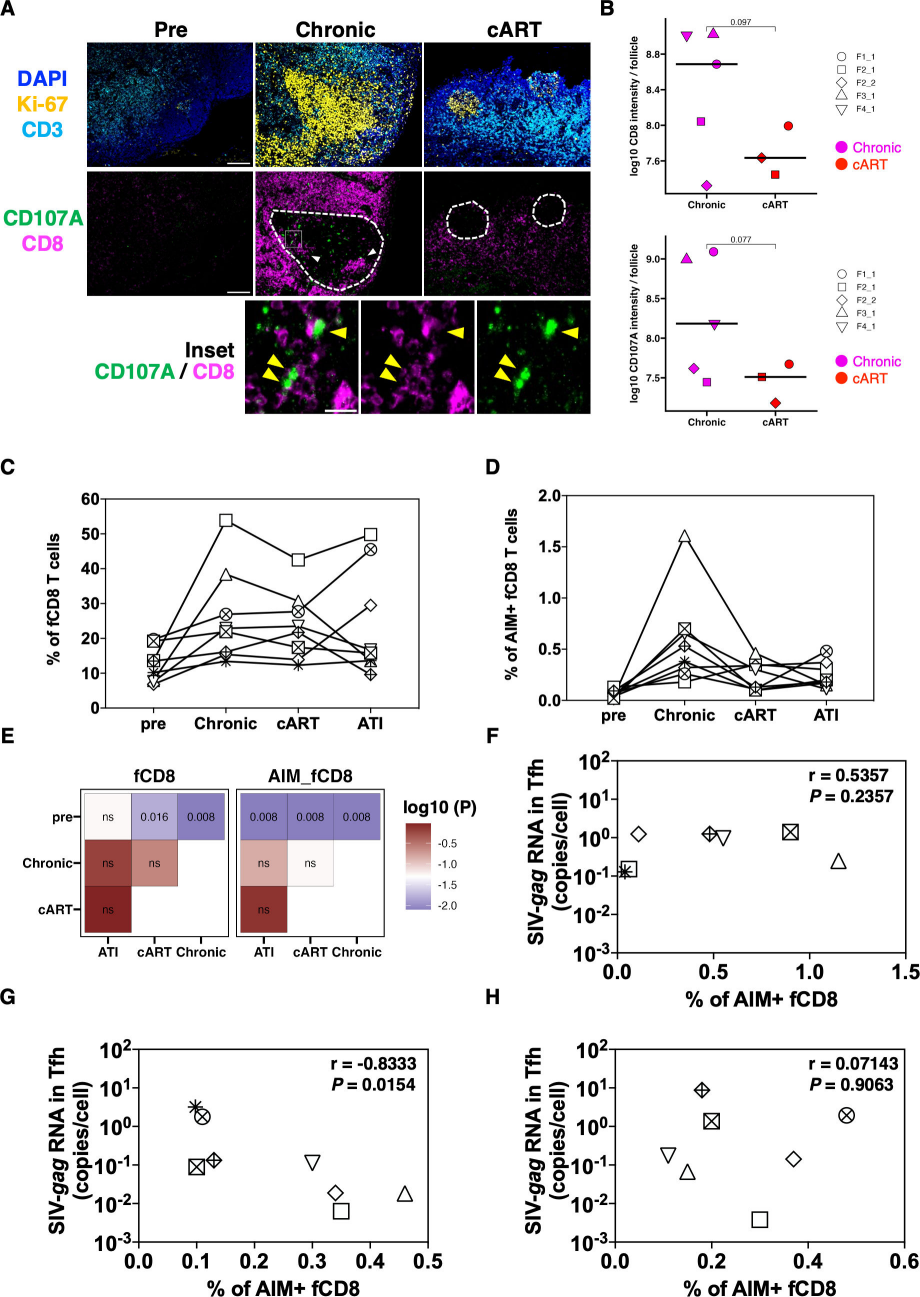
Changes in the frequency of fCD8 T cells and correlation between fCD8 T cells and SIV-*gag* RNA. (**A**) Representative immunohistochemistry data showing follicle structure (top, which includes copies of corresponding images from Fig. 1D) and CD107A and CD8 containing (middle) cells in the lymph node. The bottom part indicates the enlarged image of the inset region in the chronic stage. To indicate the relative position of follicles, adjacent serial sections stained for Ki-67 were used to mark the follicles in CD107A and CD8 staining. Arrows indicate the CD107A positive cells. (**B**) The fluorescence intensity of CD8 per follicle (top) and CD107A per follicle (bottom) are shown. Symbols indicate the microscope field and follicles obtained from one macaque. Crossbars indicate the median value. *P*-values calculated using the student’s *t*-test are shown. (**C, D**) The frequency of fCD8 T cells in memory CD8 T cells (**C**) and SIV-Gag-specific AIM^+^ fCD8 T cells (**D**) were analyzed by cell sorter. (**E**) *P*-values calculated using the pairwise Wilcoxon test are shown as a heatmap. (**F–H**) Correlations between SIV-*gag* RNA and SIV-Gag-specific AIM^+^ fCD8 T cells in the progressors are shown. LNs were biopsied at the chronic phase (**F**), cART (**G**), and ATI (**H**). SIV-*gag* RNA in Tfh at each time point was measured by qRT-PCR. The frequency of SIV-Gag-specific AIM^+^ fCD8 T cells was analyzed using a cell sorter. The correlation was calculated using Spearman’s rank correlation test. Fcd8, follicular CD8; LNs, lymph nodes; Tfh, follicular helper T cells; cART, combined antiretroviral therapy; AIM, activation-induced markers.

Previous studies have revealed that the number of fCD8 T cells present in follicles increases due to HIV/SIV infection and that fCD8 T cells have high killing activity (17–20, 30). Therefore, the intrafollicular distribution of fCD8 T cells was examined by immunofluorescence staining of lymphoid tissue sections before and after SIV infection. To mark the follicles, we used adjacent serial sections for Ki-67^+^ cell staining (Fig. 1D; Fig. S3). CD8 T cells were found not only outside the follicle but also in the follicle, where Ki-67^+^ cells accumulated during the chronic infection phase (Fig. 2A). As reported for HIV-infected LN (30), clusters of CD8^+^ cells were observed inside the follicles (Fig. 2A, arrowhead). Fluorescent intensity of CD8 signals in follicles tended to be higher in the chronic phase than under cART, but this was not observed in pre-infection samples (Fig. 2A top), supporting the accumulation of CD8 T cells in the follicle during the course of infection. Interestingly, some CD8^+^ cells co-localized with CD107A (Fig. 2A, inset), which is known to be a marker of cytotoxic activity and is also used as an AIM. Quantification of the fluorescence intensity of CD107A signals in follicles tended to be higher in the chronic phase than under cART (Fig. 2B bottom), supporting the accumulation of CD8 T cells with cytolytic properties in the follicle. These results suggest that fCD8 T cells with cytolytic properties accumulate in follicles, especially during the chronic infection phase.

**Fig 3:**
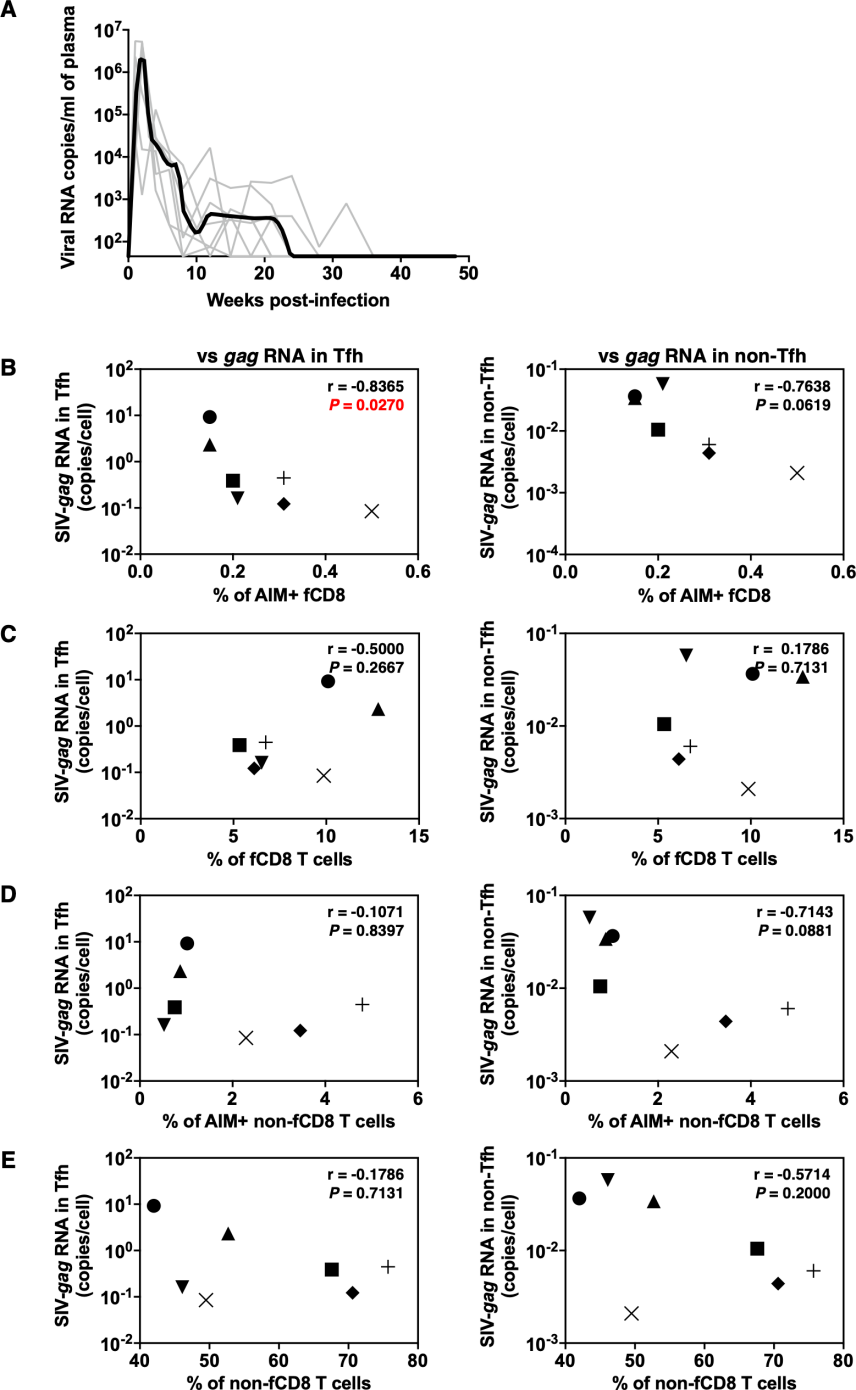
Correlation of SIV-*gag* RNA and the subpopulation of CD8 T cells in the natural controller. (**A**) Plasma viral SIV-*gag* RNA copies of natural controllers (*N* = 7) are plotted. Plasma viral RNA was measured by qRT-PCR. Gray lines show plasma viral RNA copies of each monkey. The black line shows the median. (**B, C, D, E**) Correlation of SIV-*gag* RNA in Tfh (left) or non-Tfh (right) cells and subpopulations of CD8 T cells (B, SIV-Gag-specific AIM^+^ fCD8 T cells; C, bulk fCD8 T cells; D, SIV-Gag-specific AIM^+^ non-fCD8 T cells; E, bulk non-fCD8 T cells) in the natural controllers. The target populations are shown as the *x*- or *y*-axes. The correlation was calculated using Spearman’s rank correlation test. Statistically significant *P* values are shown in red. fCD8, follicular CD8; Tfh, follicular helper T cells; AIM, activation-induced markers.

To quantify bulk and antigen-specific fCD8 T cells more accurately, we performed flow cytometric analysis. To capture the SIV-specific responses, we utilized two AIMs (4–1BB and CD107A). In our previous study, AIM^+^ cells defined by CD107A^high^ and 4–1BB^high^, but not by CD107A^low/total^ and 4–1BB^low/total^, contained functional CD8^+^ T cells and showed better correlation with disease biomarkers (31). We decided to utilize this gating limited to high MFI. Total lymphocytes were stimulated for 4 h using the Gag overlapping peptide of SIVmac239, and AIM^+^ fCD8 T cells expressing 4–1BB^high^ and/or CD107A^high^ fCD8 T cells were analyzed as SIV-Gag-specific CD8 T cells (Fig. S2C). Bulk fCD8 T cells accumulated in the follicle in the chronic infection phase, consistent with the immunofluorescence staining data; however, cART did not significantly affect the frequency (Fig. 2C and E left). In contrast, SIV-Gag-specific AIM^+^ fCD8 T cells tended to increase in the chronic infection phase and then decrease with cART; however, no significant differences were observed (Fig. 2D and E right). Furthermore, to investigate whether SIV-Gag-specific fCD8 T cells contribute to viral control in the progressor group, we examined the correlation between the proportion of SIV-*gag* RNA in Tfh cells and SIV-Gag-specific AIM^+^ fCD8 T cells in each treatment phase. There was no significant correlation between the two parameters (cell-associated SIV-*gag* RNA vs AIM^+^ fCD8 T cells) during the chronic infection phase or after cART interruption (Fig. 2F and H). However, in cART, a negative correlation was observed (Fig. 2G). This suggests that SIV-Gag-specific AIM^+^ fCD8 T cells may contribute to the control of viral replication in Tfh cells under cART in the progressor. Hence, these results suggest that SIV-Gag-specific fCD8 T cells in the LNs contribute to the regulation of SIV RNA production.

### Potential contribution of SIV-Gag-specific AIM^+^ fCD8 T cells to viral control in the natural controller group

Among the 15 SIV-infected macaques, 7 macaques naturally controlled the PVL below the detection limit by 40 wpi, and we designated these macaques as natural controllers (NC) (Fig. 3A). To determine whether SIV-Gag-specific fCD8 T cells in the NC group contributed to viral control, we examined the relationship between the amount of cell-associated SIV-*gag* RNA in Tfh and the proportion of each population of fCD8 T cells among memory CD8 T cells. A significant inverse correlation was observed between the proportion of SIV-Gag-specific AIM^+^ fCD8 T cells and cell-associated SIV-*gag* RNA in Tfh cells, but not in non-Tfh cells (Fig. 3B). In contrast, there was no correlation between the proportion of bulk fCD8 T cells and cell-associated SIV-*gag* RNA expression in Tfh cells or in non-Tfh cells (Fig. 3C). Furthermore, there was no correlation between the proportions of non-fCD8 T cells, AIM^+^ non-fCD8 T cells, and cell-associated SIV-*gag* RNA in Tfh cells or in non-Tfh cells (Fig. 3D and E). This suggests that in the NC group, SIV-Gag-specific AIM^+^ fCD8 T cells may contribute to the control of SIV RNA production in Tfh cells.

### Quantitative and qualitative differences in SIV-Gag-specific AIM^+^ fCD8 T cells in the NC and progressor groups

In both the NC and cART groups, there was an inverse correlation between the proportion of SIV-Gag-specific AIM^+^ fCD8 T cells and the amount of SIV cell-associated SIV-*gag* RNA in Tfh cells. In the NC group, PVL remained below the detection limit without treatment, whereas in the progressor group, PVL rebound was observed in all progressors when cART was interrupted. Therefore, we thought that there might be quantitative and qualitative differences in SIV-Gag-specific AIM^+^ fCD8 T cells in the NC and progressor groups (chronic and cART), and the following analysis was performed.

**Fig 4:**
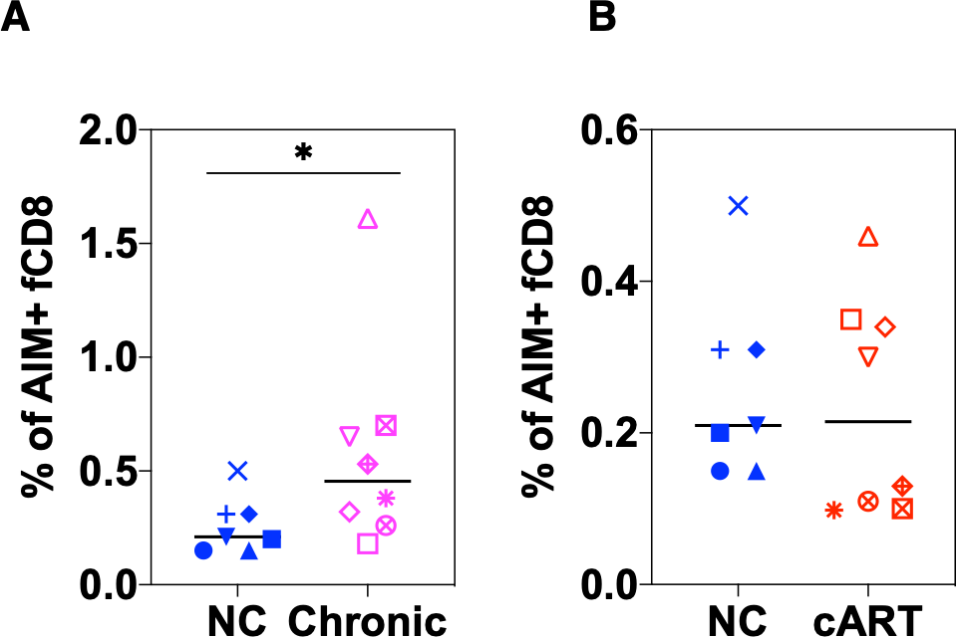
Comparison of SIV-Gag-specific AIM^+^ fCD8 and setpoint PVL in different phases. (**A, B**) Comparison of frequency of SIV-Gag-specific AIM^+^ fCD8 T cells between natural controllers and progressors at chronic phase (**A**) and after cART (**B**). *P*-values were calculated using the Mann–Whitney test. *P < 0.05. fCD8, follicular CD8; cART, combined antiretroviral therapy; PVL, plasma viral load; AIM, activation-induced markers.

First, the proportion of SIV-Gag-specific AIM^+^ fCD8 T cells was compared, and there was a significant increase of the frequency of SIV-Gag-specific AIM^+^ fCD8 T cells in the chronic infection phase in the progressor group compared to the NC group (Fig. 4A). However, there was no quantitative difference between the NC and progressor groups under cART (Fig. 4B), suggesting that quantitative differences in SIV-Gag-specific AIM^+^ fCD8 T cells did not explain the difference between the latter two groups.

**Fig 5:**
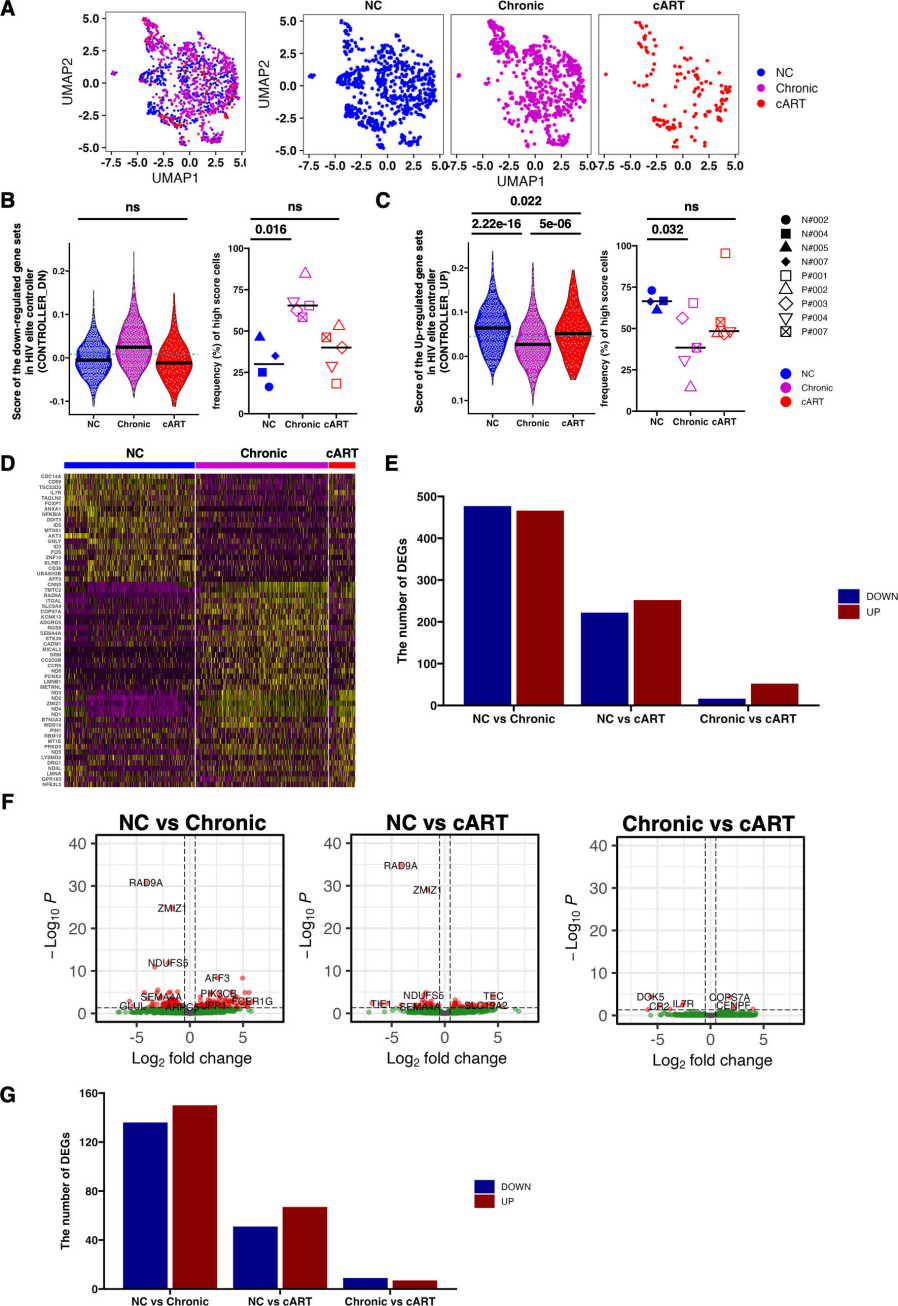
Single-cell RNA-seq analysis of SIV-Gag-specific AIM^+^ fCD8 T cells in three infection stages. (**A**) UMAP plot showing the integrated scRNA-seq data of AIM^+^ fCD8 T cells. Colors indicate groups of NC, Chronic, and cART. Merged (leftmost) image and individual groups. (**B**) Violin plot (left) shows the score of “CONTROLLER_DN(M4540)” in each cell of each group. The black crossbar indicates the median value in each group. The blue dotted line indicates the median value of all groups. The dot plot (right) shows the frequency (%) of cells showing the high score. A high score is defined as a value above the median value of all groups (above the dotted line). Crossbars indicate the median of each group. (**C**) Violin plot (left) shows the score of “CONTROLLER_UP(M4539)” in each cell of each group. The black crossbar indicates the median value in each group. The blue dotted line indicates the median value of all groups. The dot plot (right) shows the frequency (%) of cells showing high scores. A high score is defined as a value above the median value of all groups (above the dotted line). Crossbars indicate the median of each group. *P*-values were calculated using the Mann–Whitney test. (**D**) Heatmap showing relative expression of DEGs in each group. The top 20 DEGs from each group were selected for the plot. (**E**) The number of DEGs in the indicated comparisons is shown as a bar plot. The number of upregulated and downregulated genes are indicated by blue and red, respectively. (**F**) Volcano plot showing the DEGs of indicated comparisons from pseudo-bulk data. (**G**) The number of DEGs in indicated comparisons in (**F**) are shown as a bar plot. The number of upregulated and downregulated genes is indicated by blue and red, respectively. fCD8, follicular CD8; cART, combined antiretroviral therapy; DEGs, differentially expressed genes; AIM, activation-induced markers.

To delineate the qualitative differences in SIV-Gag-specific fCD8 T cells between NC and progressors (chronic and cART), we performed single-cell RNA-seq (scRNA-seq) of SIV-Gag-specific AIM^+^ fCD8 T cells. As a premise, we examined the possible contribution of MHC allele frequencies between two groups (NC and progressors); however, we could not find any biased allele distribution in either group (Tables S5 and S6). SIV-Gag-specific AIM^+^ fCD8 T cells were sorted from LNs at each treatment stage, and scRNA-seq was performed using the 10× Genomics platform. After quality filtering, we obtained transcriptome of 1403 AIM^+^ fCD8 T cells (NC = 633 cells, chronic = 641 cells, cART = 129 cells) from a total of 14 samples (NC = 4, chronic = 5, cART = 5) (Fig. 5A). To validate these data sets, we examined controller gene signatures (32), which were defined by the comparison of gene expression profiles of HIV-1 Gag tetramer-positive CD8 T cells in human PBMCs from viremic/elite controllers and progressors. The gene signature (CONTROLLER_UP), which is upregulated in HIV controllers, was increased in NC. Conversely, the gene signature (CONTROLLER_DOWN) increased in the HIV progressor group and decreased in the NC group (Fig. 5B and C, violin plots). These data suggest that our data set potentially reflects the difference between the controller and the progressor in HIV-infected individuals. These results were further validated at the individual macaque level, at which the frequency of cells with higher gene signature values than the overall median as the threshold value was calculated per macaque. A significant difference was found between NC and chronic, but no difference was found between NC and cART (Fig. 5B and C, swarm plots).

To extract the transcriptomic features of each group, we examined the differentially expressed genes (DEGs) for each of the three groups: NC, chronic, and cART stage. Characteristic gene expression patterns were observed in each process (Fig. 5D). Particularly, in NC, the expression of the activation marker *CD38*, resting or stem-like memory marker *IL7R*, and tissue retention marker *CD69* was upregulated (Fig. 5D). Intergroup comparison of the number of DEGs revealed that the number of DEGs between NC vs chronic was the highest, whereas that of chronic vs cART was the lowest (Fig. 5E). Consistently, the intergroup comparison of DEGs by pseudo-bulk analysis, in which the expression counts in all cells were summed for each macaque, showed similar results as in the scRNA-seq data (Fig. 5F and G).

Collectively, these results suggest a considerable transcriptomic difference of SIV-Gag-specific AIM^+^ fCD8 T cells between NC and Chronic groups, and SIV-Gag-specific AIM^+^ fCD8 T cells under cART have an intermediate gene expression profile.

### Cluster-wise analysis of scRNA-seq (NC vs cART)

**Fig 6:**
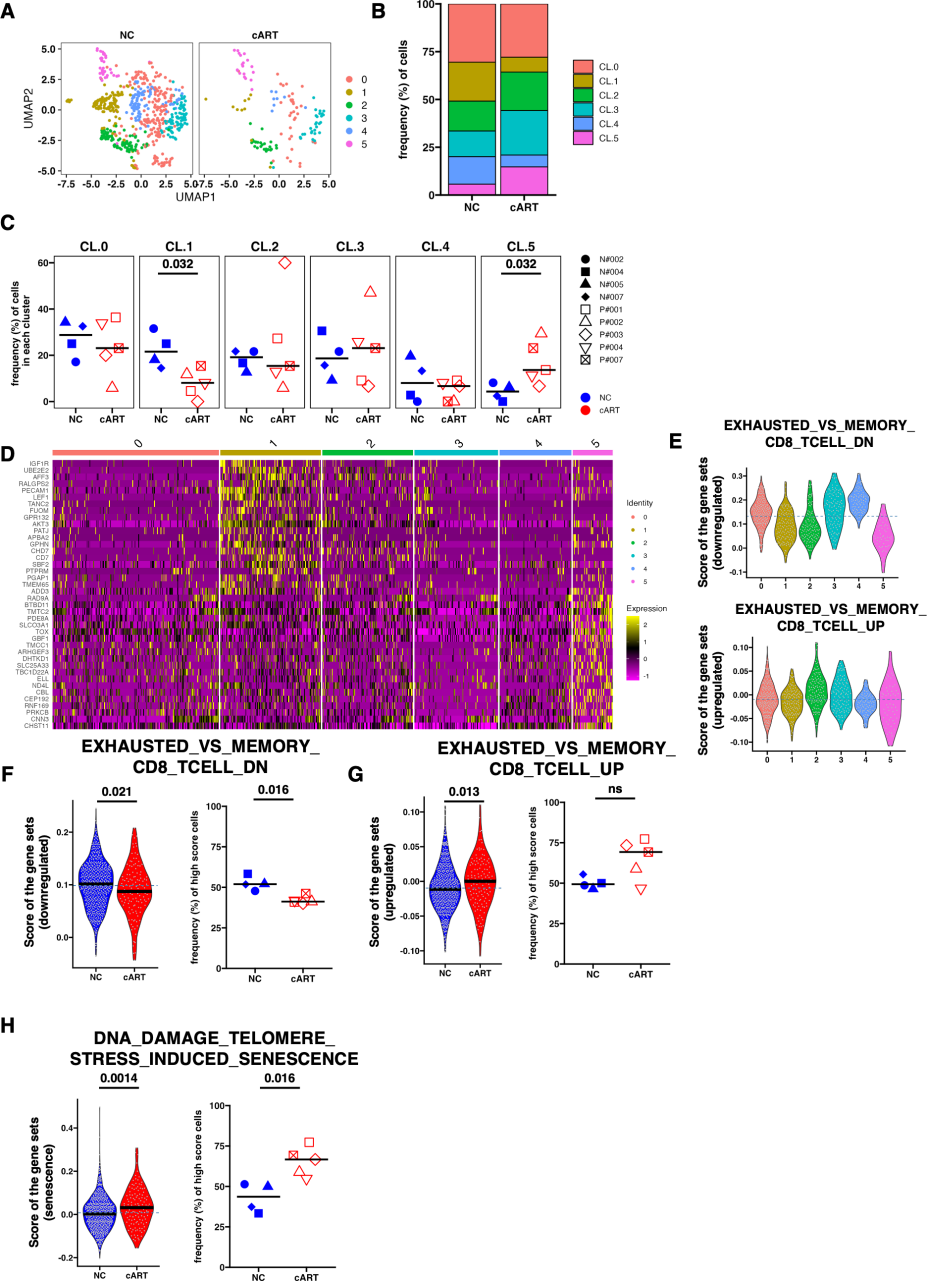
Transcriptomic comparison of SIV-Gag-specific AIM^+^ fCD8 of controller vs that of cART. (**A**) UMAP showing clustering of SIV-Gag-specific AIM^+^ fCD8 T cells in the NC and cART groups. The numbers indicate the cluster number as defined using Seurat. (**B**) Relative cluster frequency in each group. Colors indicate the cluster number, as defined by Seurat. (**C**) Dot plot showing the number of cells per macaque in each cluster. Colors indicate the group (NC or cART), and shapes indicate individual macaques. (**D**) Heatmap showing the relative expression of differentially expressed genes in each cluster. The top 20 upregulated DEGs from CL.1 and CL.5 were selected for the plot. (**E**) Violin plot (left) shows the score of upregulated and downregulated gene signatures in exhausted CD8^+^ T cells in each cell type of each group. (**F**) Violin plot (left) shows the score of “EXHAUSTED_VS_MEMORY_CD8_TCELL_DN (M5841)” in each cell of each group. Black cross bars indicate the median values in each group. The blue dotted line indicates the median value of all groups. The dot plot (right) shows the frequency (%) of cells with high scores. A high score was defined as a value above the median value of all groups (above the dotted line). The cross bars indicate the median for each group. (**G**) Violin plot (left) shows the score of “EXHAUSTED_VS_MEMORY_CD8_TCELL_UP (M5838)” in each cell of each group. Black cross bars indicate the median values in each group. The blue dotted line indicates the median value of all groups. The dot plot (right) shows the frequency (%) of cells with high scores. A high score was defined as a value above the median value of all groups (above the dotted line). The cross bars indicate the median for each group. (**H**) Violin plot (left) shows the score of “DNA_DAMAGE_TELOMERE_STRESS_INDUCED_SENESCENCE (M27191)” for each cell in each group. Black cross bars indicate the median values in each group. The blue dotted line indicates the median value of all groups. The dot plot (right) shows the frequency (%) of cells with high scores. A high score was defined as a value above the median value of all groups (above the dotted line). The cross bars indicate the median for each group. *P*-values were calculated using the Mann–Whitney test (**C, F, G, H**). fCD8, follicular CD8; cART, combined antiretroviral therapy; AIM, activation-induced markers.

To delineate the qualitative differences between the NC and cART groups in detail, the cells were separated into six clusters (CL 1–6) (Fig. 6A). A comparison of cluster frequencies showed a trend toward the enrichment of CL1 and CL4 in NCs and, conversely, less enrichment of CL5 in NCs (Fig. 6B). The quantification of the CL frequency in each macaque revealed that CL1 was significantly higher in the NC group than in the chronic group, while CL5 was significantly higher in the cART group (Fig. 6C). Given the assumption that SIV-Gag-specific AIM^+^ fCD8 T cells in NC are effective in viral suppression, properties such as CL1 would be advantageous, and properties such as CL5 would be unfavorable.

DEGs between CL1 and CL5 showed that the expression of *TOX*, a representative marker gene of exhausted memory CD8 T cells in chronic infection (33–37), was elevated in CL5, suggesting that more exhausted cells may accumulate in cART than in NC (Fig. 6D). The signatures downregulated in exhausted cells were reduced in CL5 cells (Fig. 6E), further suggesting the exhausted characteristics of CL5. Indeed, the signatures up- and downregulated in exhausted cells were increased or decreased in cART, respectively (Fig. 6F and G).

Next, to evaluate the cytotoxic activity of cells in each group, we compared the two profiles for cytotoxic activity and found no significant differences at the individual level although there was an overall trend toward higher cytotoxic activity in the NCs (Fig. S4A). Interestingly, *GZMB* expression was lower in cART, whereas *GZMK*, a marker of CD8 T cells in inflammation condition (38), tended to be lower in NCs, suggesting that immunosenescence may proceed in cART than in NCs (Fig. S4B). Therefore, we examined the senescence-related gene scores and found that the DNA damage-induced senescence score was significantly increased by cART (Fig. 6H). In addition, scores using *TOX* and *GZMK* were also increased in cells under cART (Fig. S4C). In contrast, the higher expression of *CD69*, *IL7R*, and *CD38* in the NCs suggests that they may retain a more tissue-localized and activated nature (Fig. 5D). Therefore, we analyzed the two gene signatures of virus-specific activation and tissue retention, which were increased in HIV-specific CD8 T cells in LNs (30). The results showed that both signatures were reduced in the cART group compared to the NC group (Fig. S4D). Consistently, scores using the tissue retention signature genes *CD69* and *CD103* were higher in the NCs (Fig. S4E).

## DISCUSSION

In this study, we used the NC and cART treatment models of chronic persistent SIV infection to analyze the immunological factors that contribute to the regulation of latent viruses in LNs. First, among macaques rectally infected with SIVmac239, we observed a group of individuals in which the amount of virus in the blood could be spontaneously controlled below the detection limit (NCs) and a group of individuals in which chronic persistent infection occurred (progressors). The macaques with chronic persistent infection showing a clear setpoint were treated with cART therapy, a three-drug combination of FTC/TDF/DTG. Within a few weeks, the virus level in the blood was completely reduced to below the detection limit. Furthermore, when the drug was withdrawn, a rebound in the PVL was observed in all individuals within a few weeks, settling at a setpoint similar to the pre-cART setpoint. This phenomenon closely resembles the clinical picture, suggesting that this is an excellent animal model for curative therapy, as it reflects many features of real clinical practice. It also has the advantage of allowing for an efficient analysis of the controller population, as approximately half of the infected population will be controllers. In addition, we showed that in the cynomolgus macaque SIV chronic persistent infection model, as in humans and rhesus macaques, there is an accumulation of Tfh and SIV cell-associated SIV-*gag* in Tfh associated with chronic persistent SIV infection. Besides, while it goes without saying that monitoring immune responses in LNs in HIV/SIV is important, the search for surrogate markers in peripheral blood is essential for clinical practice. With regard to the quantification of the number of SIV-infected cells with provirus, analysis of provirus levels by the IPDA method showed that provirus levels in Tfh correlated strongly (Fig. 1I, *R* = 0.84, *P* = 0.0006) with cell-associated SIV-*gag* RNA levels. Furthermore, the same method was used to measure the amount of provirus in PBMCs, which showed a strong correlation with the amount of cell-associated SIV-*gag* RNA in Tfh cells (Fig. S5A, *R* =0.85, *P* < 0.0001). These results suggest the possibility of monitoring the number and longitudinal change of SIV-infected cells with intact provirus in lymphoid tissues by the IPDA method using peripheral blood by optimizing the primer/probe design.

The results of this study not only support that our cART model of SIV infection in cynomolgus macaques is clinically relevant but also provide a new basis for extending the knowledge of SIV infection obtained to date from the crab-eating macaque model to human HIV.

Recently, Petrovas et al. have shown, using human and monkey models, that the increased accumulation of CD8 T cells in GCs, especially CXCR5-positive fCD8 T cells, during the chronic phase, is important for virus control; their properties are markedly altered by SIV infection and a decline in multifunctionality when assessed by cytokine production capacity, while granzyme and perforin, which are involved in cytotoxic activity, are highly maintained (17, 18). In addition, a comparative analysis of peripheral blood, thoracic lymph vessels, and lymphoid tissue in healthy subjects and HIV-1-positive individuals by Betts et al. showed that memory T cells in lymphoid tissue exhibit a CD69-positive CD103-positive tissue-type CD8 memory T-cell (Trm) phenotype and that this Trm subset is important for virus control (39, 40).

In the present study, we first demonstrated that in NC and under cART but not in untreated progressors, the frequency of SIV-Gag-specific fCD8 T cells defined by AIM was inversely correlated with cell-associated SIV-*gag* RNA in Tfh. Although these inverse correlations do not necessarily mean the direct control of viral replication by SIV-Gag-specific fCD8 T cells, SIV-Gag-specific fCD8 T cells may contribute to the suppression of SIV-infected cells in LNs. Alternatively, the lack of SIV antigen in the absence of viral replication during cART or natural controller leads to a decrease in immune activation and, therefore, fewer activated CD8 T cells present in the LNs as previously suggested (17, 41). Consistent with the latter possibility, we also observed more AIM^+^ fCD8 T cells in the progressors than in the controllers or under cART (Fig. 4). While the inverse correlations were only observed within groups (controller or under cART). Ferrand-Martinez et al. reported that the accumulation of fCD8 T cells may be specific to the pathogenic infection model. This conclusion was drawn by comparing the African green monkey (AGM), a non-pathogenic infection model, with the rhesus macaque, a pathogenic infection model. This finding appears to contradict the hypothesis that fCD8 T cells contribute to the control of SIV replication. However, AGMs are natural hosts for SIV and do not exhibit the inflammatory phenotype observed in pathogenic models. Additionally, it has been proposed that in AGMs, natural killer cells are primarily responsible for controlling viral replication within lymph nodes (42), indicating a potentially distinct mechanism of viral control compared to that seen in rhesus and cynomolgus macaques.

Second, scRNA-seq of SIV-Gag-specific AIM^+^ fCD8 T cells suggested that there are considerable differences of gene expression profiles between NC and chronic and that AIM^+^ fCD8 T cells in cART have an intermediate gene expression profile. This is not surprising as chronic and cART are longitudinal samplings of the same individuals in the first place, and NC is a different population; therefore, chronic and cART will be similar. However, the rebound observed in all individuals when cART was interrupted suggests that SIV-Gag-specific fCD8 T cells under cART may be qualitatively different from that under NC.

In addition, the gene signature (CONTROLLER_UP) that was increased in the HIV control group was not significantly different between NC and cART, suggesting that cART has a gene expression profile intermediate between that of NC and chronic. Consistently, the number of DEGs detected in the comparison of NC vs chronic was greater than NC vs cART. Moreover, longitudinal observations of HIV-infected individuals have shown that cART treatment alone does not restore functional abnormalities because of the maintenance of the expression of some immune checkpoint molecules even under chronic-phase cART treatment (43). Notably, one of the non-controller macaques (P001) exhibited a particularly high CONTROLLER_UP score, especially under cART. Although the precise mechanism is unclear, one possibility is that this macaque maintained elevated levels of fCD8 T cells and had the lowest SIV-*gag* RNA levels in Tfh cells, suggesting that it possesses traits similar to those of a controller. The transcriptome of SIV-Gag-specific AIM^+^ fCD8 T cells in the NC group showed high tissue retention and a higher trend of cytotoxic activity as observed in HIV-specific CD8 T cells of the HIV controller (30), reflecting the nature of the controllers in HIV. In contrast, in cART cells, in addition to the low levels of the above signatures, cellular exhaustion and senescence signatures were high. These data may indicate that the senescent state of HIV-specific CD8 T cells proceeds under cART and that cellular exhaustion may also occur. Recently, Collins et al. have consistently shown that HIV-specific CD8 T cells in controller LNs proliferate in an antigen stimulation-dependent manner and have high tissue retention and cytotoxic activity (30). On the contrary, Statzu et al. recently demonstrated the dispensableness of SIV-specific fCD8 T cells to control the reservoir size by CD8 depletion in cART-treated SIV-infected rhesus macaques (44). However, their model was based on acute SIV infection, and cART was initiated early but not in the chronic stage of infection. Further studies are required to clarify the effects of cART timing on SIV-specific fCD8 T-cell-mediated reservoir control.

SIV-specific CD8 T cells were not directly detected in the present study, and the analysis was based on AIM^+^ cells. The AIM assay, which has become widely used in studies on SARS-CoV-2-specific T cells (45), has also been used to capture HIV Gag-specific CD8 T cells. In CD8 T cells, it has been reported to correlate with IFNγ-released before and after cART (46). An inherent weakness of the AIM assay is that, in the analysis of antigen-specific CD8 T cells using tetramers, inactive tetramer-positive cells that cannot be detected by AIM have been reported (47), suggesting that AIM^+^ cells can only detect a fraction of SIV-specific CD 8T cells. In addition, because the AIM assay is usually accompanied by overnight stimulation, it is necessary to consider stimulus-dependent changes in T-cell properties, especially in gene expression analysis. We previously reported that the AIM assay works even with brief stimulation (48). To minimize stimulus-dependent changes in gene expression as much as possible, we kept the stimulation as short as possible (4 h) in our gene expression analysis. Consequently, validation using the reference signature obtained from HIV Gag tetramer-positive CD8 T cells showed that the signature that was increased in the HIV controller was also increased in our AIM-based analysis (Fig. 6).

In this study, we used only the overlapping peptide of SIV mac239 Gag, not Env or other SIV-derived antigens, as stimulating antigens. For example, analysis of the EC rhesus monkey suggests the importance of extensive CTL responses not only to Gag but also to Nef and other viral proteins (49, 50). Thus, further investigations are required to understand the entire pool of SIV-specific CD8 T responses. However, among HIV-specific CTLs, Gag-specific CTLs are known to exhibit stronger antiviral activity (51). Furthermore, Collins et al. reported that among the dominant epitope analyzed by tetramers, all but one of the 19 controllers with various HLA types was the Gag epitope (30), suggesting that targeting Gag in the controller likely targets the primary functional HIV-specific CD8 T cells involved in viral suppression. Furthermore, the sample size of our analysis was insufficient. For scRNA-seq, in particular, we have not been able to secure a sufficient number of macaques experimentally. As a result, the power to detect statistically significant differences is limited. Nevertheless, the fact that our results are consistent with those reported for HIV, despite the small sample size, may reflect an essential phenomenon related to HIV/SIV infection. Although we discussed the mechanisms of SIV viral replication suppression by comparing the three groups, it remains possible that the control mechanisms vary among individuals within the seven macaques in the controller groups. One possibility is that this variation is linked to MHC alleles or haplotypes, as seen with protective alleles in HIV. However, our limited MHC class I analysis did not reveal any association with SIV control. Furthermore, the contribution of non-Gag-specific CD8 T cells warrants further investigation in future studies.

Finally, we examined potential peripheral biomarkers to capture changes in fCD8 T cells in the LNs (peripheral fCD8 T cells). We first examined the frequency of CXCR5-positive CD8 T cells in the peripheral blood, which correlated with the frequency of fCD8 T cells in LNs in specimens subjected to cART (Fig.S6A and B). However, this correlation was not observed in untreated samples. To explore the molecules that correlate more precisely, we gated peripheral CXCR5^+^ memory CD8 T cells with CXCR3/PD-1/ICOS and found that the frequencies of several memory CD8 T-cell subsets defined by CXCR3^+^CXCR5^+^, PD-1^+^CXCR5^+^, and ICOS^−^CXCR5^+^ correlated better and more strongly with the frequencies of fCD8 T cells using cART. In particular, the subset defined by ICOS^−^CXCR5^+^ showed the strongest correlation (Fig. S6C, *R* =0.67, *P* = 0.046), suggesting that the ICOS^−^CXCR5^+^ memory CD8 T-cell subset in the peripheral blood may be considered pfCD8 T cells, as originally assumed. In contrast, the frequencies of ICOS^+^CXCR5^+^ memory CD8 T cells were correlated in untreated specimens, suggesting changes in the characteristics of fCD8 T cells during the course of treatment. As mentioned above, in our cynomolgus macaque model, we have shown that the amount of cell-associated SIV-*gag* RNA in Tfh correlates with the amount of provirus measured by IPDA in the peripheral blood, indicating that peripheral blood may be used to assess the intact provirus in LNs indirectly. In addition, a detailed subset analysis of the frequency of CXCR5^+^ peripheral memory CD8 T cells to examine fCD8 T-cell counterparts in peripheral blood showed that the combination of ICOS and CXCR5 may be used to assess fCD8 T-cell frequency in peripheral blood. Although this surrogate marker is only used to determine the frequency of bulk fCD8 T cells, previous reports suggest that HIV-specific CD8 T cells in the peripheral blood and LNs may function in a coordinated manner. For example, analysis of HIV-specific CD8 T cells in LNs and peripheral blood of HIV controllers suggested that HIV-specific CD8 T cells in LNs and PBMCs are common at the clonotype level and show similar phenotypes in terms of their cytotoxic activity and proliferative potential (30).

In summary, in the present study, we demonstrated that the frequency of SIV-Gag-specific fCD8 T cells, defined by AIM, is inversely correlated with cell-associated SIV-*gag* RNA in Tfh cells under NC and cART, using NC and cART treatment models with chronic persistent SIV infection. Furthermore, qualitative differences in SIV-Gag-specific fCD8 T-cell levels between NC- and cART-treated macaques may contribute to the suppression of latently SIV-infected cells in LNs. To confirm the potential interactions between fCD8 T and Tfh cells within the lymphoid tissue, further high-dimensional analyses, such as spatial transcriptomics, focusing on the relative positioning of these cells would be highly informative. Our results and previous findings suggest that it is difficult to completely restore the function of SIV-Gag-specific fCD8 T cells in LNs using cART alone. In other words, improving these phenotypes under cART may lead to a cure and is expected to provide clues for establishing new curative therapies.

## MATERIALS AND METHODS

### Animal experiments and cART treatment

Fifteen cynomolgus macaques (*Macaca fascicularis*) were housed at the Tsukuba Primate Research Center (TPRIC), NIBION under the supervision of veterinarians in charge of the animal facility. All macaques tested negative for SIV, simian type D retrovirus, simian T-cell lymphotropic virus, simian foamy virus, Epstein–Barr virus, cytomegalovirus, and B virus and were intrarectally inoculated with SIVmac239 up to three times at 5 × 10^4^ TCID50. The week before the plasma viral load was detected was defined as 0 week of infection. Eight of the 15 macaques received combination antiretroviral therapy (cART) from 68 to 96 wpi. cART contains dolutegravir (DTG; 10 mg kg^−1 ^d^−1^), tenofovir (TFV; 60 mg kg^−1^ d^−1^), and emtricitabine (FTC; 120 mg kg^−1^ d^−1^). The cART was subcutaneously inoculated once daily.

### LN biopsy and LN mononuclear cell preparation

Blood was collected under anesthesia with EDTA as an anticoagulant. PBMCs and plasma were isolated using Ficoll-Paque PLUS (GE Healthcare, Buckinghamshire, UK) as previously described (52). The LNs were collected via longitudinal biopsy. LN was homogenized and filtrated through a 0.7-µm filter to obtain LMNC. PBMC or LNMC were stocked in FBS supplemented with 10% FBS at −150°C until use. For IHC, LN were washed with ice-cold R10 and fixed with 10% neutral buffered formalin (FUJIFILM Wako) overnight. Fixed tissue samples were embedded in paraffin blocks.

### Flow cytometric analysis and sorting

Frozen LNMC was thawed, washed with RPMI1640 medium (Sigma-Aldrich, St. Louis, MO, USA) supplemented with 10% FBS (Sigma-Aldrich), 100 U/mL of penicillin, and 100 mg/mL streptomycin (Sigma-Aldrich; hereafter referred to as R10) and treated with 1 mL benzonase (50 U/mL; Merck, Darmstadt, Germany) in R10 for 30 min at 37°C. Cells were stimulated with SIVmac_239_ overlapping Gag peptides (NIH AIDS Reagent Program) at a concentration of 1 µg/mL in the presence of anti-CD107a and anti-4–1BB for 4 h. After 4 h incubation, cells were incubated with FcR blocking reagent Human (Miltenyi Biotec) for 10 min at 4°C. The dead cells were then stained with LIVE/DEAD Fixable Blue Dead Cell Stain Kit for 5 min at room temperature. Antibodies used in this study are listed in Table S1. Chemokine receptors CXCR5 and CCR7 were stained for 15 min at 37°C followed by staining of the cell surface markers with Anti-CD3, Anti-CD4, Anti-IgG, Anti-IgD, Anti-CD19, Anti-CD95, Anti-PD-1, Anti-CD20, Anti-CD8, Anti-CD28, Anti-CD14, Anti-CD16, Anti-CD56, and Anti-TCRgd together with TotalSeq Anti-Human Hashtag Antibody (Biolegend) for 15 min at room temperature. The cells were then washed and suspended in PBS. Since in our previous study, AIM^+^ cells defined by CD107A^high^ and 4–1BB^high^, but not by CD107A^low/total^ and 4–1BB^low/total^, contained functional CD8 T cells and showed better correlation with disease biomarkers (31), we decided to utilize this gating limited to high MFI for AIM^+^ gating. Accordingly, six subsets (Tfh, non-Tfh, AIM^+^ fCD8 T cells, AIM^−^ fCD8 T cells, AIM^+^ Non-fCD8 T cells, and AIM^−^ Non-fCD8 T cells) were gated, as shown in Fig. S2, and sorted using a BD FACSymphony S6 cell sorter (BD Biosciences). The data were analyzed using FlowJo v. 10.8.1. The frequency of the occurrence in each population was calculated.

### Measurement of plasma viral RNA loads

Plasma viral RNA loads were measured using a highly sensitive quantitative real-time RT-PCR approach, as described previously (52). Briefly, viral RNA in plasma was purified using the MagNA PureCompact Nucleic Acid Isolation Kit (Roche Diagnostics, Rotkreuz, Switzerland). Real-time RT-PCR was performed using the QuantiTec Probe RT-PCR kit (Qiagen, Germany) and a Light Cycler 480 thermocycler (Roche Diagnostics, Rotkreuz, Switzerland). The limit of detection was 45 viral RNA copies/mL.

### vRNA measurement

The Tfh and Non-Tfh-cell populations were sorted as described above. Each cell population was centrifuged, the cell pellet was resuspended in 750 µL of ISOGEN II (NipponGene), and cellular RNA was isolated following the manufacturer’s protocol. The SIV-*gag* RNA was amplified by qRT-PCR using SuperScript III Platinum One-Step qRT-PCR Kit (Invitrogen) using the 5′-FAM-CTTC(dP)TCAGT(dK)TGTTTCACTTTCTCTTCTGCG-BHQ1-3′ probe and primers 5′-GTCTGCGTCAT(dP)TGGTGCATTC-3′ and 5′-CACTAG(dK)TGTCTCTGCACTAT(dP)TGTTTTG-3′. The number of SIV-*gag RNA* copies was normalized to the number of sorted cells. If the number of sorted cells was less than 50, we did not measure SIV-*gag RNA*.

### Intact proviral DNA assay

Intact proviral SIV copy numbers were measured by droplet digital PCR (ddPCR) using IPDA (26). Total genomic DNA was extracted from Tfh cells and PBMC using a QIAamp DNA mini kit (QIAGEN) following the manufacturer’s protocol. The ddPCR reactions were carried out using a QX200 Droplet Digital PCR system and ddPCR Multiplex Supermix (Bio-Rad) following the manufacturer’s instructions. To determine intact proviruses, the *pol*/*env* and 2-LTR/*env* sites were amplified using 900 nM of each primer and 250 nM of each probe. To quantify internal control and calculate the DNA-shearing index, two sites of the RPP30 gene with the same distance as *pol* and *env* sites were amplified using 500 nM of each primer and 250 nM of each probe. Droplets were generated using a QX200 Droplet generator (Bio-Rad), and reactions were performed with the following cycles: 95°C for 10 min, 50 cycles of (94°C for 30 s, 56°C for 2 min), 98°C for 10 min. Droplets were read by the QX200 Droplet reader (Bio-Rad). The copies of intact provirus per million cells were calculated with each copy number of *pol*/*env*, 2-LTR/*env,* and RPP30 and corrected with DNA-shearing index. The primers and probes used for IPDA are listed in Table S2.

### Immunohistochemistry

Lymphoid tissue sections for immunohistochemistry were obtained from lymphoid tissue over time before and after SIV infection. All IHC assays were performed using a Leica BOND-RXm platform (Leica). Formalin-fixed, paraffin-embedded LN were sectioned at 3 µm. Antigen retrieval was performed using BOND Epitope Retrieval Solution 2 (prediluted, pH 9.0) for 20 min at 99°C. Specimens were stained with a titrated amount of unconjugated primary antibodies for 45 min at RT and washed with 1× BOND Wash Solution (Leica). The specimens were incubated with appropriate secondary antibodies for 45 min at RT and washed again. The specimens were then stained with titrated amounts of conjugated antibodies for 45 min. After washing, the nucleoli were stained with nuclear stain for 5 min at RT, and the slides were mounted with Fluoromount G (Thermo Fisher). The antibodies used for the immunohistochemistry are listed in Table S3. Image quantification was performed by ImageJ (version 1.54 g). In each section for Ki-67 staining, boundaries of follicles were manually defined based on the expression pattern of Ki-67 and CD20 (Fig. 1D; Fig. S3). In the sections for CD8/CD107A staining (Fig. 2A and B), follicles were indirectly defined by the adjacent serial sections for Ki-67 staining (Fig. 1D).

### Single-cell RNA-seq library preparation and sequencing

scRNA-seq libraries were prepared from the SIV-Gag-specific AIM^+^ fCD8 T cells sorted as described above. Sorted cells were centrifuged at 1,000 × *g* for 5 min, washed once with PBS, centrifuged at 2,000 × *g* for 5 min, resuspended in ~17 µL of PBS, and used as input for the chromium 3′-kit (10× Genomics, Pleasanton, CA, USA) beads/macaque. The scRNA-seq library was produced according to the 10× Genomics User Guide for Chromium Next GEM Single-Cell 3′ Reagent Kits v3.1. Libraries were combined and read using an Illumina NovaSeq 6000 platform (AZENTA, Burlington, MA, USA).

### scRNA-seq analysis and visualization

Raw sequence reads were analyzed using Cell Ranger 6.1.2 (10 × Genomics) with the human crab-eating macaque reference genome (version 6.0) and the corresponding GTF file. The output matrixes from the Cell Ranger were analyzed using *Seurat 5.0* (53, 54). The raw gene expression matrixes were converted into Seurat objects. Each datum was separated, the quality was first controlled by the total number of genes detected (more than 200 genes), the frequency of mitochondrial genes (cells showing less than 10% of the mitochondrial gene frequency), and the ribosomal genes (cells showing more than 0.05% of the ribosomal gene frequency).

After quality control, Seurat objects from each macaque were merged and then integrated using the *Seurat::SelectIntegrationFeatures* (nfeatures = 3000), *PrepSCTIntegration*, *FindIntegrationAnchors* (normalization.method=“SCT”), and *IntegrateData* functions. Integrated sea objects were normalized and clustered using the *Seurat::ScaleData*, *RunPCA*, *FindNeighbors*, *FindClusters*, and *RunUMAP* functions. Plots were generated using the *Seurat::DimPlot*, *FeaturePlot*, *VlnPlot*, and *scCustomize::FeaturePlot_scCustom* functions. DEGs were identified using *Seurat::FindMarkers* by comparing cluster-high (CL-high) and CL-low samples with default parameters. Among the DEGs of the CL-high or CL-low samples, 20 genes were selected from those with the lowest *P*-values, and heatmaps were generated from the selected genes using *Seurat::DoHeatmap*. Module scores were calculated using the *Seurat::AddModuleScore* function with the selected genes, as shown in Table S4. For the pseudo-bulk analysis, scRNA-seq data were converted to the bulk RNAseq count matrix. Briefly, the expression counts in all cells were summed for each macaque. The resulting count matrix was used to extract DEGs by *DESeq2* (55).

### Statistical analysis

Experimental variables were analyzed using Spearman’s correlation, Wilcoxon signed-rank test, and Mann–Whitney *U* test. *P* value <  0.05 was considered statistically significant. GraphPad Prism statistical analysis software (version 9; GraphPad Software, San Diego, CA, USA) or R/Bioconductor (R 4.2.1/Bioconductor 3.16) was used.

## Data Availability

Data have been deposited to NCBI/ENA/DDBJ under accession number PRJDB17186.

## SUPPLEMENTAL MATERIAL

Supplemental text jvi.00812-24-s0001.docx Supplemental methods and legends.

Supplemental material jvi.00812-24-s0002.pdf Figures S1 to S6; Tables S1 to S6.

ASM does not own the copyrights to Supplemental Material that may be linked to, or accessed through, an article. The authors have granted ASM a non-exclusive, world-wide license to publish the Supplemental Material files. Please contact the corresponding author directly for reuse.
