# Supplementary material for: The quality of SIV-specific fCD8 T cells limits SIV RNA production in Tfh cells during antiretroviral therapy
Source: J Virol. 2024 Dec 6;99(1):e00812-24. doi: 10.1128/jvi.00812-24 (PMC11784340; doi:10.1128/jvi.00812-24)
Supplement: Supplemental text — Supplemental methods and legends. [file jvi.00812-24-s0001.docx]

# Supplemental Material

# Supplemental Methods

## MHC allele typing

MHC alleles were identified using next-generation sequencing (NGS)-based methods as developed by Dr. David O'Connor's group (https://dholk.primate.wisc.edu/_webdav/dho/grants/mhc_contract/web_portal/@files/prototype/genotyping/genotyping/protocols/all_macaque_mhc_exon2.html). A total of 60 MHC-target NGS libraries (amplicon of 4 primer sets targeting class I region from 15 macaques) were pooled together for the MiSeq run. A total of 18,750,645 paired-end sequence pass filter reads were obtained. Read1 and read2 from a paired-end sequence reads were merged using bbmerge. An average of 248,084 sequences were obtained per amplicon after merging read1 and read2. Merged sequences were aligned to Mafa-A1, A2, A3, A4, A5, A6, and A8 and Mafa-B reference alleles (release version 3.13.0.0, 2024-07 IPD-MHC NHP database) using bbmap. Coverage was counted using samtools. The top two ranked alleles with at least 1,000 reads of coverage were selected for each Mafa-A locus, while the top six ranked alleles with at least 1,000 reads of coverage were selected for the Mafa-B locus.

# Supplemental Figure legends

## Figure S1. Plasma viral RNA copies and reservoirs of SIV-infected cynomolgus macaques.

(**A**) Plasma viral RNA copies of fifteen SIV-infected cynomolgus macaques were plotted on the graph. Plasma viral RNA was measured by qRT-PCR. Red lines indicate progressors, and blue lines indicate controllers. (**B**) SIV Gag RNA copies in Tfh and Non-Tfh at the untreated chronic-phase (wk67 for progressors, wk123 for controllers) were analyzed by qRT-PCR. Shapes indicate the individual macaques. Statistical significance was calculated using the Wilcoxon matched-pairs signed-rank test; ns: no significance; Tfh, follicular helper T cells.

## Figure S2. Gating strategy for cell sorting.

(**A**) Representative gating for the CD4 or CD8 T cells. After gating on single cells, lymphocytes were gated by FSC-A and SSC-A. Subsequently, the CD3^+^ alive population was further negatively gated by lineage markers and then positively gated by CD4 and CD8 expression. (**B**) Memory CD4 T cells were defined based on CD28 and CD95 expression, then Tfh and Non-Tfh were gated by PD-1 and CXCR5 antibodies. (**C**) CD28/CD95 antibodies were used to gate memory subsets of CD8 T cells. Among the memory CD8^+^ T cells, fCD8 and Non-fCD8 T cells were gated based on CCR7 and CXCR5 expression. SIV Gag-specific AIM^+^ fCD8 or AIM^+^ non-fCD8 were defined as 4-1BB^high^ and/or CD107A^high^ cells. Fcd8, follicular CD8; AIM, activation-induced markers; Tfh, follicular helper T cells.

## Figure S3. Immunohistochemical staining of Tfh.

(**A**) Representative immunofluorescence staining of follicles in LN of the chronic stage of SIV-infected cynomolgus macaque. The individual staining (grayscale), as indicated by the header and the merged image (color), are shown. Header text colors indicate the pseudo-colors in the merged image. The area shown by the white line indicates a border between the inside and outside of the follicle. (**B**) Representative follicular areas defined by Ki-67 (Green) and CD20 (magenta) are shown. The white line indicates a border between the inside and outside of the follicle. (**C**) Representative image of colocalization of PD-1 and CD3. The three right images are enlarged images of the inset in the leftmost image. The arrowhead indicates the CD3, but not PD-1, positive cell; Tfh, follicular helper T cells.

## Figure S4. scRNA-seq analysis of cytolytic, activation, and tissue retention functions.

(**A**) Violin plot (leftmost) shows the score of "GOBP_CELL_KILLING(M15770)" in each cell of each group. The black crossbar indicates the median value in each group. The blue dotted line indicates the median value of all groups. The dot plot (right-left) shows the frequency (%) of cells showing high scores. A high score is defined as a value above the median value of all groups (above the dotted line). Crossbars indicate the median of each group. Violin plot (right-left) shows the score of "GOCC_CYTOLYTIC_GRANULE(M25840)" in each cell of each group. The black crossbar indicates the median value in each group. The blue dotted line indicates the median value of all groups. The dot plot (rightmost) shows the frequency (%) of cells showing high scores. A high score is defined as a value above the median value of all groups (above the dotted line). Crossbars indicate the median of each group. (**B**) Violin plot shows the expression of GZMB (left) and GZMK (right) in each group. Crossbars indicate the median of each group. (**C**) Violin plot (left) shows the module score calculated by the expression of TOX and GZMK in each cell of each group. The black crossbar indicates the median value in each group. The blue dotted line indicates the median value of all groups. The dot plot (rightmost) shows the frequency (%) of cells showing high scores. A high score is defined as a value above the median value of all groups (above the dotted line). Crossbars indicate the median of each group. (**D**) Violin plots show the score of the "T-cell activation" signature and "Tissue retention" signature defined by Collins et al. 2023 in each cell of each group. The black crossbar indicates the median value in each group. The blue dotted line indicates the median value of all groups. The dot plot (rightmost) shows the frequency (%) of cells showing high scores. A high score is defined as a value above the median value of all groups (above the dotted line). Crossbars indicate the median of each group. (**E**) Violin plot (left) shows the module score involving tissue retention calculated by the expression of CD69 and CD103 in each cell of each group. The black crossbar indicates the median value in each group. The blue dotted line indicates the median value of all groups. The dot plot (rightmost) shows the frequency (%) of cells showing high scores. A high score is defined as a value above the median value of all groups (above the dotted line). Crossbars indicate the median of each group. *P*-values were calculated using the Mann-Whitney test.

## Figure S5. Correlation between SIV Gag RNA in Tfh and IPDA in PBMC.

SIV Gag RNA copies in Tfh cells were measured by qRT-PCR. The intact provirus in PBMC was measured using IPDA. The yellow symbols indicate the Natural Controllers. The blue, red, and green symbols indicate the progressors in the chronic, cART, and ATI phases, respectively. Correlation coefficients and *P*-value were calculated using Spearman's rank correlation test. Tfh, follicular helper T cells; IPDA, intact proviral DNA assay.

## Figure S6. Identification of peripheral counterpart of fCD8 T cells in LNs.

(**A**) A scatter plot shows the correlation between the frequency of fCD8 T cells per total memory CD8 T cells in LN and CXCR5^+^ cells in total memory peripheral CD8 T cells. Colors indicate the samples from cART-treated and non-treated macaques. The correlation coefficient and *P*-value were calculated for total samples by Spearman's correlation analysis. (**B**) Scatter plots show the correlation between the frequency of fCD8 T cells per total memory CD8^+^ T cells in LN and CXCR5^+^ cells in total memory peripheral CD8 T cells. Colors indicate samples from cART-treated (blue) and non-treated (red) macaques. The correlation coefficient and *-* value were calculated for each group by Spearman's correlation analysis. (**C**) Scatter plots show the correlation between the frequency of fCD8 T cells per total memory CD8^+^ T cells in LN versus a subset of CXCR5^+^ peripheral CD8 memory T cells. Marker (+) subset was defined by CXCR5^+^ AND one of CCR7/CD127/CD69/CD95/CXCR3/ICOS/PD-1 positive subsets. Marker (-) subsets were defined by CXCR5^+^ AND ICOS negative subset. The correlation coefficient and *P*-value were calculated by Spearman's rank correlation analysis. Fcd8, follicular CD8; LNs, lymph nodes.

# Supplemental Tables legends

## Table S1. Antibodies for flowcytometry/sorting.

List of antibodies used for the flowcytometry/sorting.

## Table S2. Primer and probes for IPDA.

Primer and probe information (sequence, flurophore and quenchers) used for IPDA.

## Table S3. Antibodies for immunohistochemistry.

List of antibodies used for the immunohistochemistry.

## Table S4. Gene signatures used for module analysis.

List of gene signature name and IDs used for module analysis.

## Table S5. Mafa-A alleles of the fifteen macaques.

List of the Mafa-A alleles of the fifteen macaques included in this study.

## Table S6. Mafa-B alleles of the fifteen macaques.

List of the Mafa-B alleles of the fifteen macaques included in this study.
