# Supplementary material for: The quality of SIV-specific fCD8 T cells limits SIV RNA production in Tfh cells during antiretroviral therapy
Source: J Virol. 2024 Dec 6;99(1):e00812-24. doi: 10.1128/jvi.00812-24 (PMC11784340; doi:10.1128/jvi.00812-24)
Supplement: Supplemental material — Figures S1 to S6; Tables S1 to S6. [file jvi.00812-24-s0002.pdf]

Supplemental Figure 1

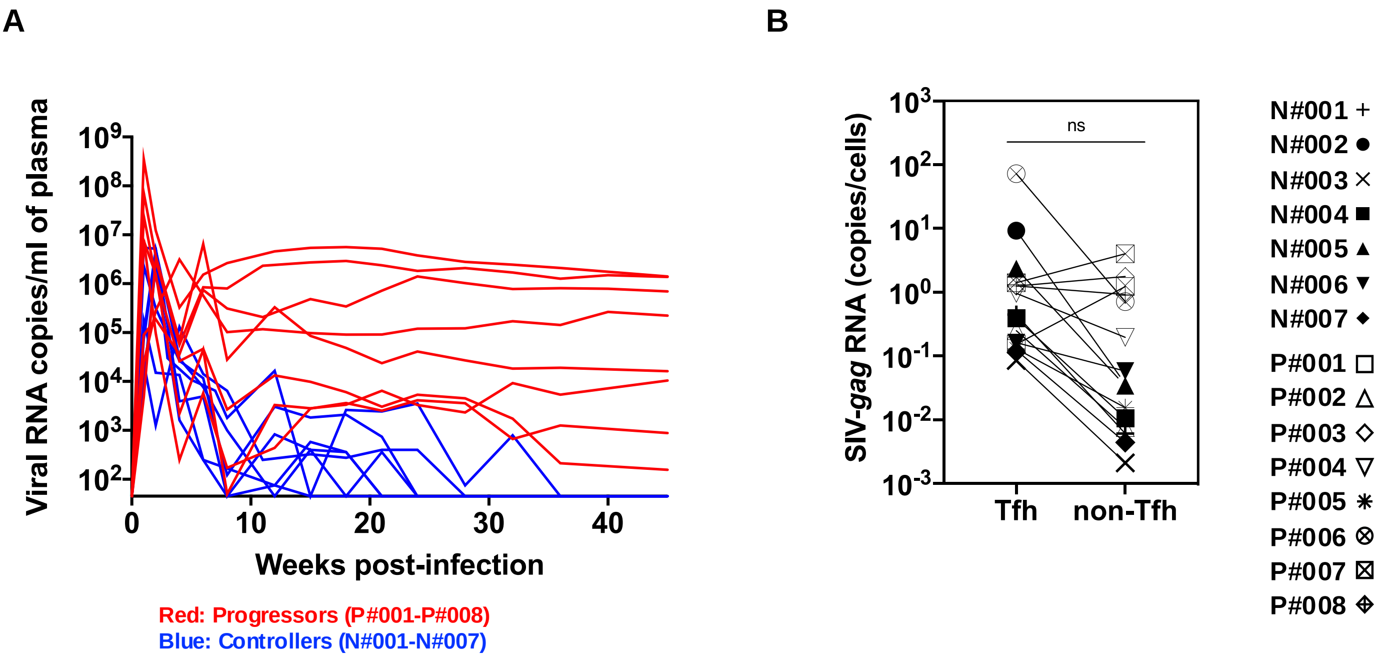

Supplemental Figure 2

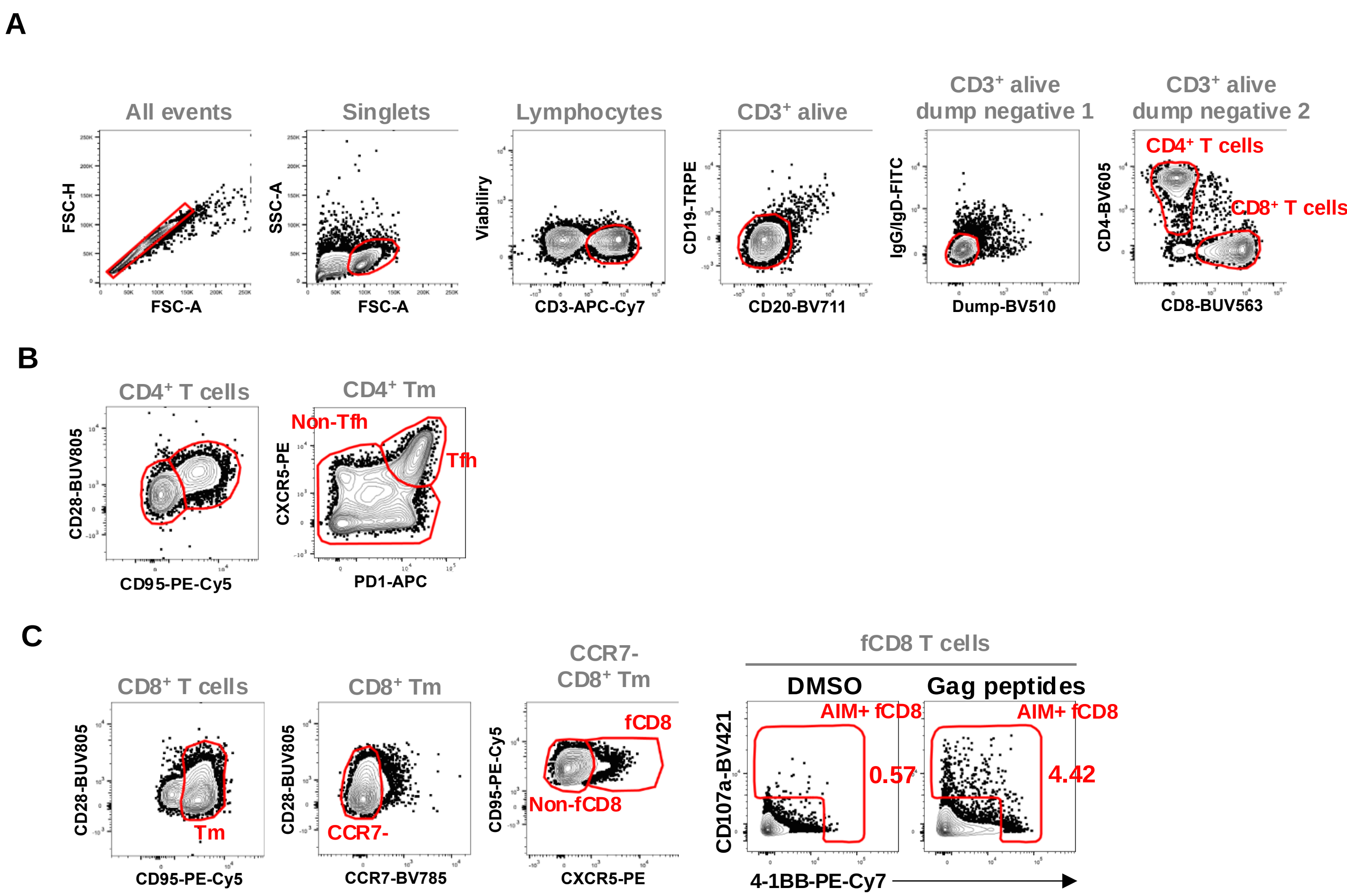

Supplemental Figure 3

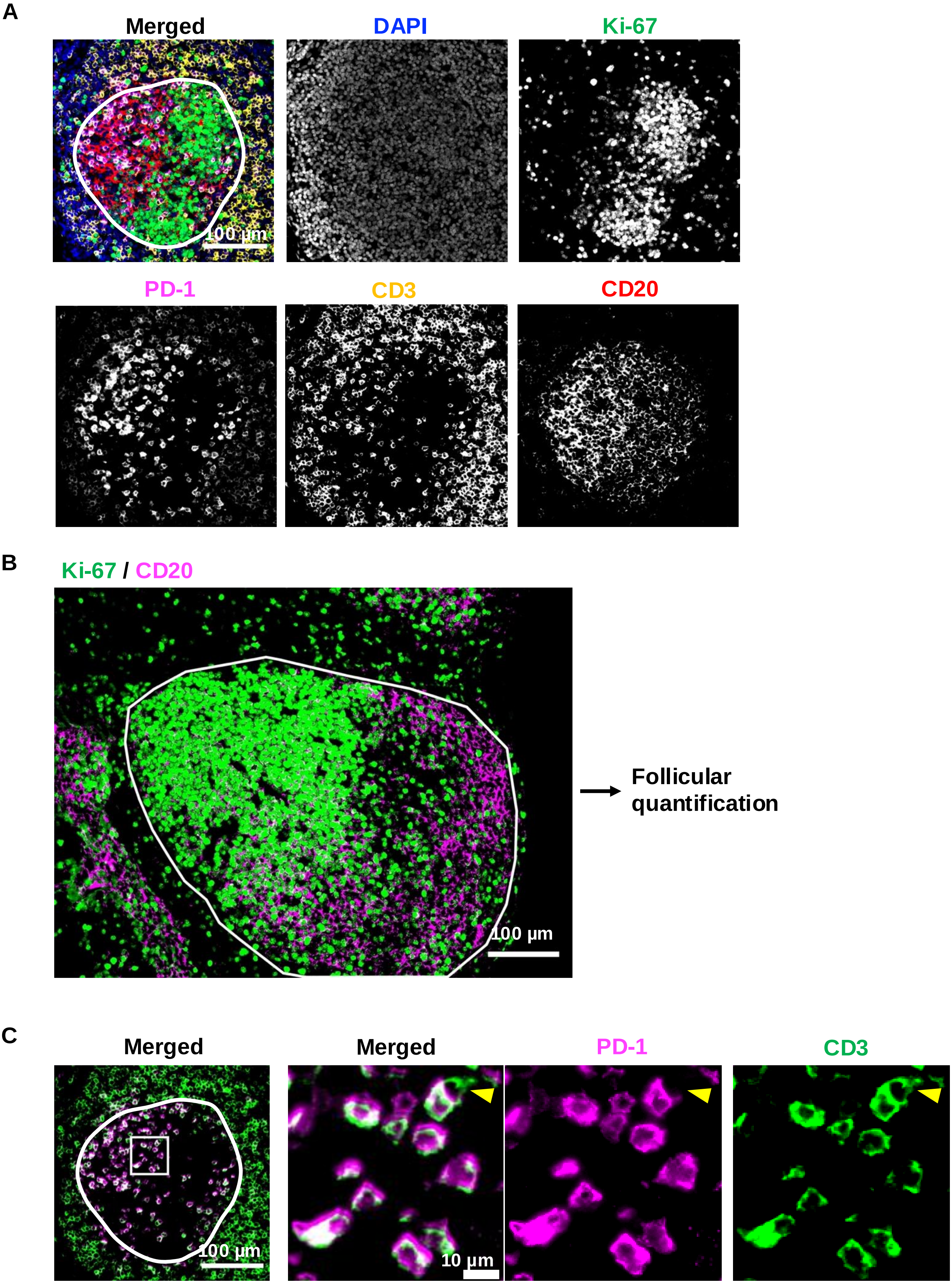

Supplemental Figure 4

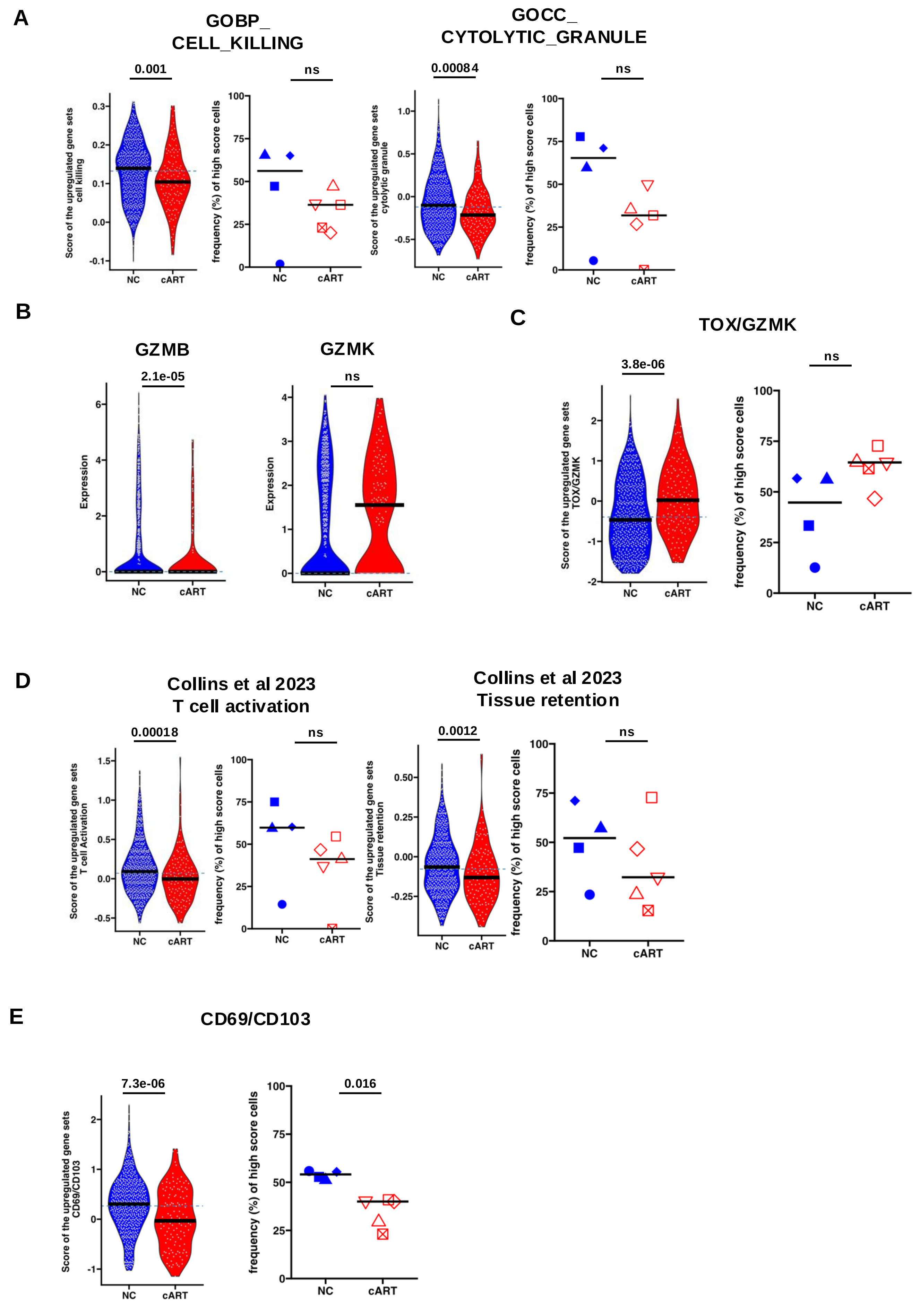

Supplemental Figure 5

A

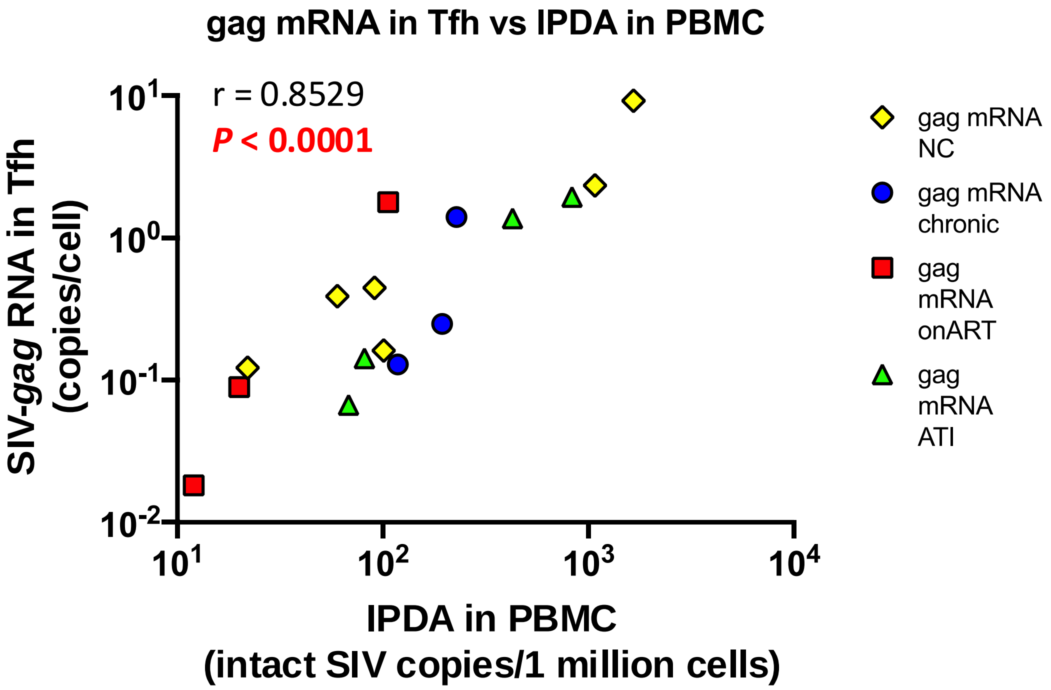

Supplemental Figure 6

A

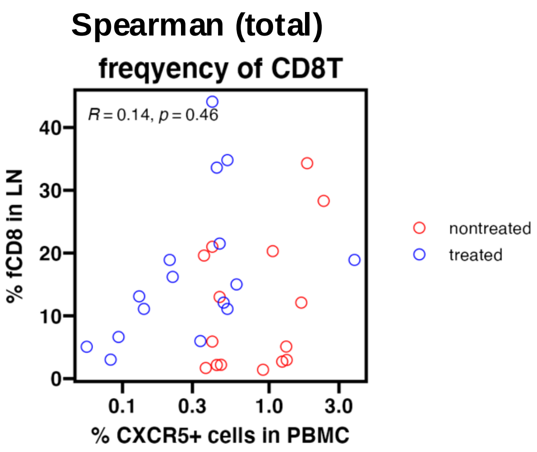

B

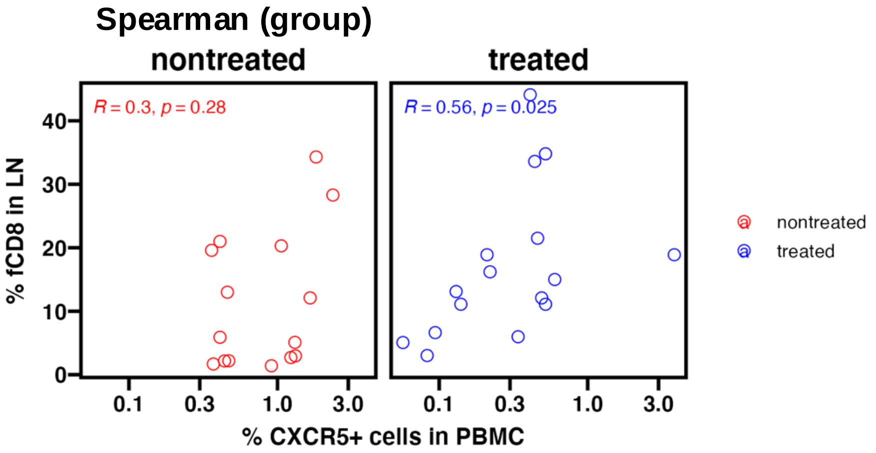

C

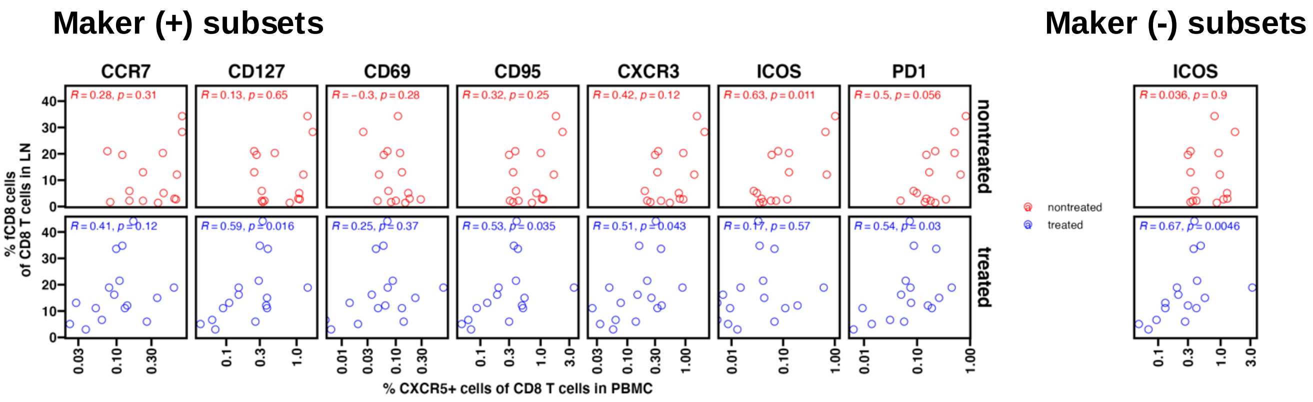

Supplemental Table 1

Antigen / target	Clone	Fluorochrome	Company	Cat#	RRID	Staining
**IgD**	poly	FITC	Southern Biotech	2030-02	not found	Surface
**IgG**	G18-145	FITC	BD Biosciences	555786	AB_396121	Surface
**CXCR5**	MU5UBEE	PE	eBioscience	12-9185-42	AB_11218894	Surface (Chemokine)
**CD19**	J3-119	ECD	Beckman Coulter	A07770	AB_2940901	Surface
**CD95**	DX2	PE-Cy5	BD Biosciences	559773	AB_397317	Surface
**4-1BB**	4B4-1	PE-Cy7	BioLegend	309818	AB_2207741	Surface
**PD-1**	EH12.2H7	APC	BioLegend	329908	AB_940475	Surface
**CD3**	SP34-2	APC-Cy7	BD Biosciences	557757	AB_396863	Surface
**CD107A**	H4A3	BV421	BD Biosciences	562623	AB_2737685	Surface
**CD4**	L200	BV605	BD Biosciences	562843	AB_2737833	Surface
**CD20**	2H7	BV711	BioLegend	302342	AB_2562602	Surface
**CCR7**	G043H7	BV785	BioLegend	353230	AB_2563630	Surface (Chemokine)
**CD8**	RPA-T8	BUV563	BD Biosciences	612914	AB_2870199	Surface
**CD28**	CD28.2	BUV805	BD Biosciences	742037	AB_2871331	Surface
**CD14**	M5E2	BV510	Biolegend	301842	AB_2561946	Surface (Dump)
**CD16**	3G8	BV510	Biolegend	302048	AB_2562085	Surface (Dump)
**CD56**	B159	BV510	BD Biosciences	740171	AB_2739924	Surface (Dump)
**TCR gamma/delta**	B1	BV510	Biolegend	331220	AB_2564275	Surface (Dump)
**LIVE/DEAD™ Fixable Blue Dead Cell Stain Kit**	n/a	n/a	Thermo Fisher Scientific	L23105	not found	Surface

Supplemental Table 2

Amplicon	Name	Sequence	Fluorophor	Quencher
SIVmac239 IPDA *pol*	polF	GCAGGGATAGAGCACACCTTTG	N/A	N/A
	polR	CTATGGTTTCTACTGAATTTGCTTGTTC	N/A	N/A
	pol intact probe	TTTCAGGTGGTGATTCA	FAM	MGB
	pol hyper probe	TAGGTGGTGATTTATT	None	MGB
SIVmac239 IPDA env	envF	CCTCAATAAAGCCTTGTGTAAAATTATC	N/A	N/A
	envR	GTTGTTATTGATTTTGTCAATCCC	N/A	N/A
	env intact probe	TGCATTACTATGAGATGC	VIC	MGB
	env hyper probe	TGCATTACTATAAAATGC	None	MGB
SIVmac239 IPDA 2LTR	239-2LTR_F	GCAGGGATAGAGCACACCTTTG	N/A	N/A
	239-2LTR_R	CTATGGTTTCTACTGAATTTGCTTGTTC	N/A	N/A
	239-2LTR_P	CCCTGGTCTGTTAGGACCCTTTCTGCTTTG	FAM	ZEN/IABkFQ
RPP30-1	RPP30-1F	AGGATGCTCCGGGAGTATGTA	N/A	N/A
	RPP30-1R	CCTGCTTGTCACCTATATAACAT	N/A	N/A
	RPP30-1 prove	TCAAGCTGGGAGACGGAAGAGTCAGT	FAM	ZEN/IABkFQ
RPP30-2	RPP30-2F	ACAGACTCACACAATTTAGG	N/A	N/A
	RPP30-2R	ACATTCATGCCACTGCACTC	N/A	N/A
	RPP30-2 probe	ACAGGGTCTCACTTTGTTGTCCA	HEX	ZEN/IABkFQ

Supplemental Table 3

Primary/secondary	Antigen / target	host	Clone	Fluorochrome	Company	Cat#	RRID
primary	Ki67	mouse	B56	Alexa 555	BD Biosciences	558617	AB_647108
primary	CD4	rabbit	EPR6855	Alexa 488	abcam	ab196372	AB_2889191
primary	CD3e	rabbit	EP449E	Unconjugated	abcam	ab52959	AB_868901
primary	PD-1	goat	polyclonal	Unconjugated	R&D Systems	AF1086	AB_354588
primary	CD8	rabbit	D8A8Y	Unconjugated	Cell Signaling Technology	85336	AB_2800052
primary	CD107A (LAMP-1)	mouse	H4A3	Unconjugated	BioLegend	328602	AB_1134259
secondary	Rabbit IgG FC	goat	polyclonal	Alexa 750	abcam	ab175735	not found
secondary	Rabbit IgG (H+L)	goat	polyclonal	Alexa 555	Invitrogen	A-21428	not found
secondary	Mouse IgG H&L	goat	polyclonal	Alexa 647	abcam	ab150115	not found
secondary	Goat IgG (H+L)	donkey	polyclonal	Alexa 647	Invitrogen	A-21447	not found
secondary	NUCLEAR STAIN FOR PHENOCYCLER (DAPI)	na	na	na	AKOYA biosciences	7000003	na

Supplemental Table 4

Systemic Name	Reference PMID	Standard name	Short Name
M4539	20890291	GSE24081_CONTROLLER_VS_PROGRESSOR_HIV_SPECIFIC_CD8_TCELL_UP	CONTROLLER_UP
M4540	20890291	GSE24081_CONTROLLER_VS_PROGRESSOR_HIV_SPECIFIC_CD8_TCELL_DN	CONTROLLER_DN
M5838	17950003	GSE9650_EXHAUSTED_VS_MEMORY_CD8_TCELL_UP	EXHAUSTED_VS_MEMORY_CD8_TCELL_UP
M5841	17950003	GSE9650_EXHAUSTED_VS_MEMORY_CD8_TCELL_DN	EXHAUSTED_VS_MEMORY_CD8_TCELL_DN
M27191	N/A	REACTOME_DNA_DAMAGE_TELOMERE_STRESS_INDUCED_SENESCENCE	DNA_DAMAGE_TELOMERE_STRESS_INDUCED_SENESCENCE
M15770	N/A	GOBP_CELL_KILLING	GOBP_CELL_KILLING
M25840	N/A	GOCC_CYTOLYTIC_GRANULE	GOCC_CYTOLYTIC_GRANULE

Supplemental Table 5

Group	Macaques	Mafa-A1	Mafa-A2	Mafa-A3	Mafa-A4	Mafa-A5	Mafa-A6
NC	N#001	Mafa-A1*060:04	NA	NA	NA	Mafa-A5*30:06	Mafa-A6*01:15
NC	N#002	Mafa-A1*089:07,Mafa-A1*052:01	NA	Mafa-A3*13:08	NA	NA	NA
NC	N#003	Mafa-A1*060:04	Mafa-A2*05:65	NA	Mafa-A4*14:12	NA	Mafa-A6*01:15
NC	N#004	Mafa-A1*032:01	NA	NA	Mafa-A4*14:12	NA	NA
NC	N#005	Mafa-A1*089:03	NA	Mafa-A3*13:08	NA	Mafa-A5*30:05	NA
NC	N#006	Mafa-A1*008:02	Mafa-A2*05:57	NA	NA	NA	Mafa-A6*01:08
NC	N#007	Mafa-A1*097:01	Mafa-A2*05:57	NA	NA	Mafa-A5*30:04	NA
Progressor	P#001	Mafa-A1*019:06,Mafa-A1*008:06	NA	Mafa-A3*13:02	Mafa-A4*14:17	NA	NA
Progressor	P#002	Mafa-A1*067:06	NA	NA	Mafa-A4*14:18	NA	NA
Progressor	P#003	Mafa-A1*041:01	NA	NA	Mafa-A4*14:18	NA	NA
Progressor	P#004	Mafa-A1*032:01,Mafa-A1*010:08	Mafa-A2*05:57	Mafa-A3*13:12	Mafa-A4*14:18	NA	NA
Progressor	P#005	Mafa-A1*066:03	Mafa-A2*05:57	Mafa-A3*13:33	Mafa-A4*14:12	NA	NA
Progressor	P#006	Mafa-A1*032:01	NA	NA	Mafa-A4*14:12	Mafa-A5*30:04	NA
Progressor	P#007	Mafa-A1*009:01	Mafa-A2*24:09	Mafa-A3*13:08	NA	Mafa-A5*30:06	NA
Progressor	P#008	Mafa-A1*010:08	Mafa-A2*05:57	Mafa-A3*13:08	NA	NA	NA

Supplemental Table 6

Group	Macaques	Mafa-B
NC	N#001	Mafa-B*047:03,Mafa-B*069:02
NC	N#002	Mafa-B*091:02,Mafa-B*013:09,Mafa-B*137:06
NC	N#003	Mafa-B*047:03,Mafa-B*101:03,Mafa-B*083:05
NC	N#004	Mafa-B*056:02,Mafa-B*017:01
NC	N#005	Mafa-B*065:03,Mafa-B*013:09,Mafa-B*161:04
NC	N#006	Mafa-B*056:02,Mafa-B*099:01
NC	N#007	Mafa-B*121:02,Mafa-B*018:01,Mafa-B*081:01
Progressor	P#001	Mafa-B*056:02,Mafa-B*017:01
Progressor	P#002	Mafa-B*144:06,Mafa-B*015:01,Mafa-B*151:02
Progressor	P#003	Mafa-B*101:05,Mafa-B*076:03,Mafa-B*153:02
Progressor	P#004	Mafa-B*030:04,Mafa-B*118:01
Progressor	P#005	Mafa-B*076:04,Mafa-B*010:01,Mafa-B*045:02
Progressor	P#006	Mafa-B*013:09,Mafa-B*144:05,Mafa-B*101:08,Mafa-B*137:06
Progressor	P#007	Mafa-B*069:02,Mafa-B*118:01,Mafa-B*144:05
Progressor	P#008	Mafa-B*056:02,Mafa-B*034:02,Mafa-B*144:03N
